# Porcine reproductive and respiratory syndrome virus N protein-mediated viral replication enhancement via interaction with host caspase-6

**DOI:** 10.1128/jvi.00163-26

**Published:** 2026-04-14

**Authors:** Dihua Zhu, Chaojun Fu, Yana Dong, Qianjun Zhang, Huixin Li, Yankuo Sun, Guihong Zhang, Heng Wang

**Affiliations:** 1 Guangdong Provincial Key Laboratory of Zoonosis Prevention and Control, College of Veterinary Medicine, South China Agricultural University 554665, Guangzhou, China; 2 Maoming Branch, Guangdong Laboratory for Lingnan Modern Agriculture, Maoming, China; 3 National Engineering Research Center for Breeding Swine Industry, South China Agricultural University 12526, Guangzhou, China; Loyola University Chicago - Health Sciences Campus, Maywood, Illinois, USA

**Keywords:** live attenuated vaccine, immune evasion, N protein, caspase-6, PRRSV

## Abstract

Porcine reproductive and respiratory syndrome virus (PRRSV) poses a persistent threat to the global swine industry, owing to its high genetic variability and immune evasion capabilities. However, the mechanisms by which PRRSV manipulates host cell apoptosis to promote its own replication and evade the host immune response remain inadequately understood. This study reveals, for the first time, a critical role for caspase-6—a cysteine-aspartic protease of the apoptotic cascade—in PRRSV infection. We demonstrated that caspase-6 specifically cleaves the viral nucleocapsid (N) protein at aspartate residue 94 (D94), generating N-terminal and C-terminal fragments that subsequently inhibit the activation and nuclear translocation of the key host transcription factor, namely, interferon (IFN) regulatory factor 3 (IRF3). This leads to a significant reduction in the expression of host IFN-β, thereby promoting viral replication. Further investigation confirmed that this cleavage site is highly conserved across different PRRSV strains, suggesting it as a potential target for the development of broad-spectrum antiviral therapeutics. Moreover, a D94A mutant virus (PRRSV-D94A), constructed using reverse genetics, exhibited significantly attenuated replication and pathogenicity. This mutant induced a more robust host antiviral immune response, characterized by markedly elevated levels of IFNs and inflammatory cytokines, indicating its potential as an ideal live attenuated vaccine candidate. This study elucidated, from the perspective of a host protease, a novel molecular mechanism by which PRRSV evades the host immune response and promotes viral replication, providing a vital theoretical basis and a new strategy for PRRSV vaccine design and antiviral drug development.

**IMPORTANCE:**

Porcine reproductive and respiratory syndrome virus (PRRSV) remains one of the most economically devastating pathogens in the swine industry, largely due to its ability to evade innate immunity and persist within its host. This study identifies, for the first time, a host–virus mechanism in which PRRSV recruits caspase-6 to cleave its N protein at a conserved D94 site, thereby suppressing type I IFN signaling and enhancing viral replication. We further demonstrate that disrupting this cleavage through the D94A mutation attenuates viral replication, enhances innate immune activation, and reduces pathogenicity in pigs. These findings not only reveal a previously unrecognized immune-evasion strategy of PRRSV but also highlight caspase-6 and the N-protein cleavage site as promising targets for host-directed antiviral interventions and rational vaccine design.

## INTRODUCTION

Porcine reproductive and respiratory syndrome virus (PRRSV) poses one of the greatest global threats to the swine industry, causing severe reproductive failures in sows, including abortions, stillbirths, weak piglets, and significant respiratory disorders characterized by increased morbidity and mortality rates (1, 2). Additionally, affected growing pigs often suffer growth retardation and increased susceptibility to secondary infections, collectively imposing substantial economic losses worldwide (3, 4). Current PRRSV control primarily relies on vaccination; however, owing to the lack of proofreading activity in the viral RNA polymerase, PRRSV exhibits a remarkably high mutation rate, resulting in rapid viral evolution and substantial antigenic variation. Consequently, existing vaccines frequently fail to induce effective cross-protection against emerging strains, underscoring an urgent need to elucidate the detailed molecular interactions between PRRSV and host immune defenses, which are critical for developing effective broad-spectrum vaccines and antiviral therapeutics.

PRRSV, belonging to the genus *Arterivirus* within the family *Arteriviridae*, is an enveloped, positive-sense, single-stranded RNA virus. Its genome, approximately 15 kb in length, encodes multiple open reading frames (ORFs) (5, 6). Among these, ORF1a and ORF1b encode nonstructural proteins (NSPs) essential for viral replication and transcription, including critical enzymes, such as RNA-dependent RNA polymerase. Structural proteins are encoded by ORF2–7, primarily including GP2a, GP3, GP4, GP5, membrane (M) protein, and N protein. GP2a, GP3, GP4, GP5, and the M protein form a glycoprotein complex on the viral surface, facilitating viral recognition and entry into host cells (7). The N protein, a core structural and functional component of the virus, binds directly to the viral genomic RNA, playing pivotal roles in genome packaging, regulation of viral replication and transcription, and modulation of virus–host immune interactions (8, 9). Notably, the PRRSV N protein has been identified as a crucial regulator of host immune responses, making it a significant target in studies aiming to elucidate viral pathogenesis and develop novel antiviral strategies (10, 11).

The host innate immune system, particularly type-I interferon (IFN) signaling pathways, constitutes the first line of defense against viral infections (12). Pattern recognition receptors (PRRs) in host cells detect viral RNA, initiating activation of transcription factors, including IFN regulatory factor 3 (IRF3) and nuclear factor-κB (NF-κB), leading to robust induction of type-I IFNs, especially IFN-β, and establishing an antiviral state (13). Despite this robust innate immune defense, PRRSV has evolved sophisticated mechanisms to evade immune surveillance effectively, among which the manipulation of programmed cell death pathways, particularly apoptosis, represents a crucial immune evasion strategy facilitating viral replication and persistence (14).

Cysteine-aspartic proteases (caspases) function as central executors of programmed cell death pathways, and increasing evidence highlights their pivotal roles during viral infections (15). Among these, caspase-6, an important effector caspase, has been proven to exert crucial host-regulatory functions during the infection processes of various viruses. For instance, during severe acute respiratory syndrome coronavirus (SARS-CoV-1) infection, the viral N protein is specifically cleaved by host caspase-6, and the resulting viral protein fragments effectively promote viral replication. Furthermore, during Zika virus and Dengue virus infections, the expression level and enzymatic activity of caspase-6 dynamically regulate the viral life cycle; similarly, human immunodeficiency virus infection also promotes viral maturation and release after the activation of caspase-6 in apoptotic host cells (16–18). These studies suggest that the involvement of host caspase-6 in regulating viral replication is a relatively common and important phenomenon. However, despite the preliminary elucidation of caspase-6’s regulatory potential in numerous viral infections and existing evidence indicating that PRRSV can influence caspase-related signaling, the specific mechanisms of individual members, such as caspase-6, in PRRSV infection still lack systematic investigation (8, 19).

Therefore, a thorough investigation into the specific function and regulatory mechanisms of caspase-6 during PRRSV infection is essential. We hypothesized that caspase-6 contributes to host antiviral defense during PRRSV infection, and that PRRSV may counteract caspase-6-mediated antiviral activity. Such a study will not only unveil the molecular basis of PRRSV’s immune evasion and pathogenic strategies but may also provide a theoretical foundation and potential targets for the development of targeted antiviral interventions.

## RESULTS

### Caspase-6 facilitates PRRSV replication

**Fig 1:**
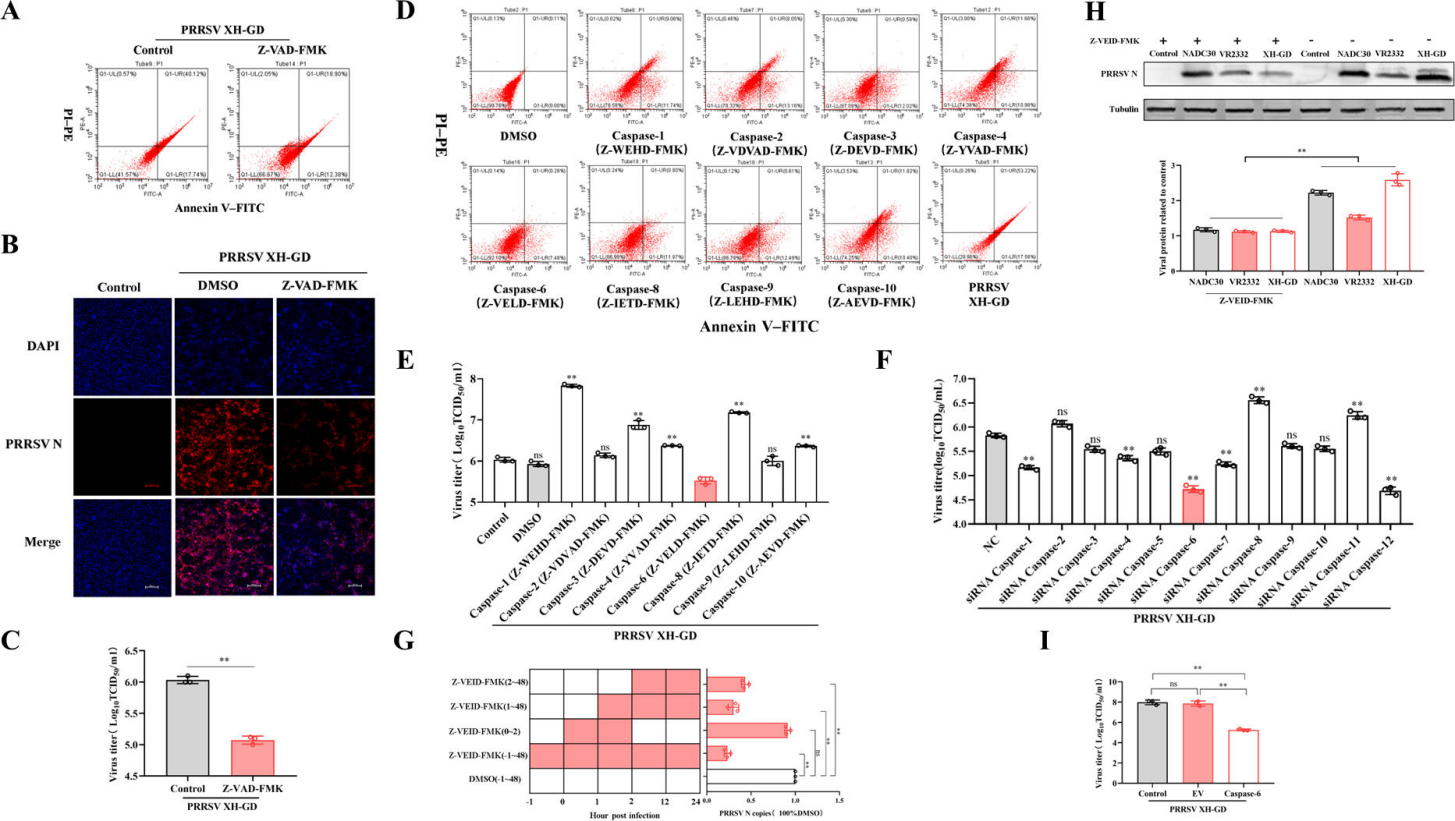
Caspase-6 inhibition restricts PRRSV replication. (**A**) Flow cytometric analysis of apoptosis in PRRSV-infected Marc-145 cells. Cells were infected with PRRSV (MOI = 1) and treated with either DMSO or the pan-caspase inhibitor Z-VAD-FMK (100 μM). At 24 h post-infection (hpi), cells were stained with Annexin V and propidium iodide (PI) and analyzed by flow cytometry. The x-axis represents Annexin V fluorescence intensity, and the y-axis represents PI fluorescence intensity. Quadrants indicate viable (Annexin V⁻/PI⁻), early apoptotic (Annexin V^+^/PI⁻), late apoptotic (Annexin V^+^/PI^+^), and necrotic (Annexin V⁻/PI^+^) cell populations. (**B**) Indirect immunofluorescence analysis of PRRSV N protein expression in Marc-145 cells infected with PRRSV and treated with DMSO or Z-VAD-FMK (100 μM). Cells were fixed at 24 hpi and immunostained using an anti-PRRSV N protein antibody. (**C**) Viral titers in the supernatants of PRRSV-infected Marc-145 cells treated with DMSO or Z-VAD-FMK, determined by TCID₅₀ assay at 24 hpi. (**D, E**) Flow cytometric analysis of apoptosis (**D**) and corresponding viral titers (**E**) in PRRSV-infected Marc-145 cells treated with individual caspase-specific inhibitors. Cells were fixed at 24 hpi and stained with Annexin V/PI for apoptosis analysis. (**F**) Viral titers in Marc-145 cells following siRNA-mediated knockdown of individual caspases. Cells were transfected with the indicated siRNAs for 24 h and subsequently infected with PRRSV (MOI = 1). Viral titers were measured at 24 hpi (*n* = 3). (**G**) Time-of-addition assay of the caspase-6 inhibitor Z-VEID-FMK in PRRSV-infected Marc-145 cells. Z-VEID-FMK was added at the indicated time points, and PRRSV N gene copy numbers in the supernatants were quantified by RT–qPCR at 24 hpi (*n* = 3). (**H**) PRRSV N protein expression levels in Marc-145 cells infected with different PRRSV strains (NADC30, VR2332, and XH-GD) in the presence or absence of Z-VEID-FMK. (**I**) Viral titers in Marc-145 cells following caspase-6 overexpression, measured at 24 hpi (*n* = 3). Data are presented as mean ± SD from three independent experiments unless otherwise indicated. Statistical significance is indicated by *P < 0.05, **P < 0.01; ns, not significant (*P* ≥ 0.05).

Although PRRSV infection can induce apoptosis in host cells, the specific mechanisms remain unclear (2, 8, 20). In this study, the pan-caspase inhibitor Z-VAD-FMK, which inhibits apoptosis (Fig. 1A), significantly restricted PRRSV replication (Fig. 1B and C). This indicated that PRRSV actively modulates the host apoptotic response to promote its own replication, suggesting a previously unrecognized link beyond simple induction of cell death. To identify which caspase is most critical for regulating PRRSV replication, we used PRRSV XH-GD as a model virus. Using siRNA-mediated knockdown and specific caspase inhibitors, we assessed their individual effects on viral replication. Our results demonstrated that the inhibition of caspase-6 had the most profound restrictive effect on PRRSV replication (Fig. 1D through F).

To further explore the role of caspase-6 in PRRSV replication, we added the specific caspase-6 inhibitor Z-VEID-FMK at different time points post-infection with PRRSV XH-GD. The inhibitor did not reduce viral replication when added concurrently with the virus, suggesting that PRRSV entry was not impaired (Fig. 1G). Furthermore, the inhibitory effect of Z-VEID-FMK on PRRSV replication was conserved across different strains (NADC30, VR2332, XH-GD) (Fig. 1H). Notably, overexpression of caspase-6 also led to an inhibition of PRRSV replication (Fig. 1I).

### Caspase-6 interacts with and cleaves the PRRSV N protein

**Fig 2:**
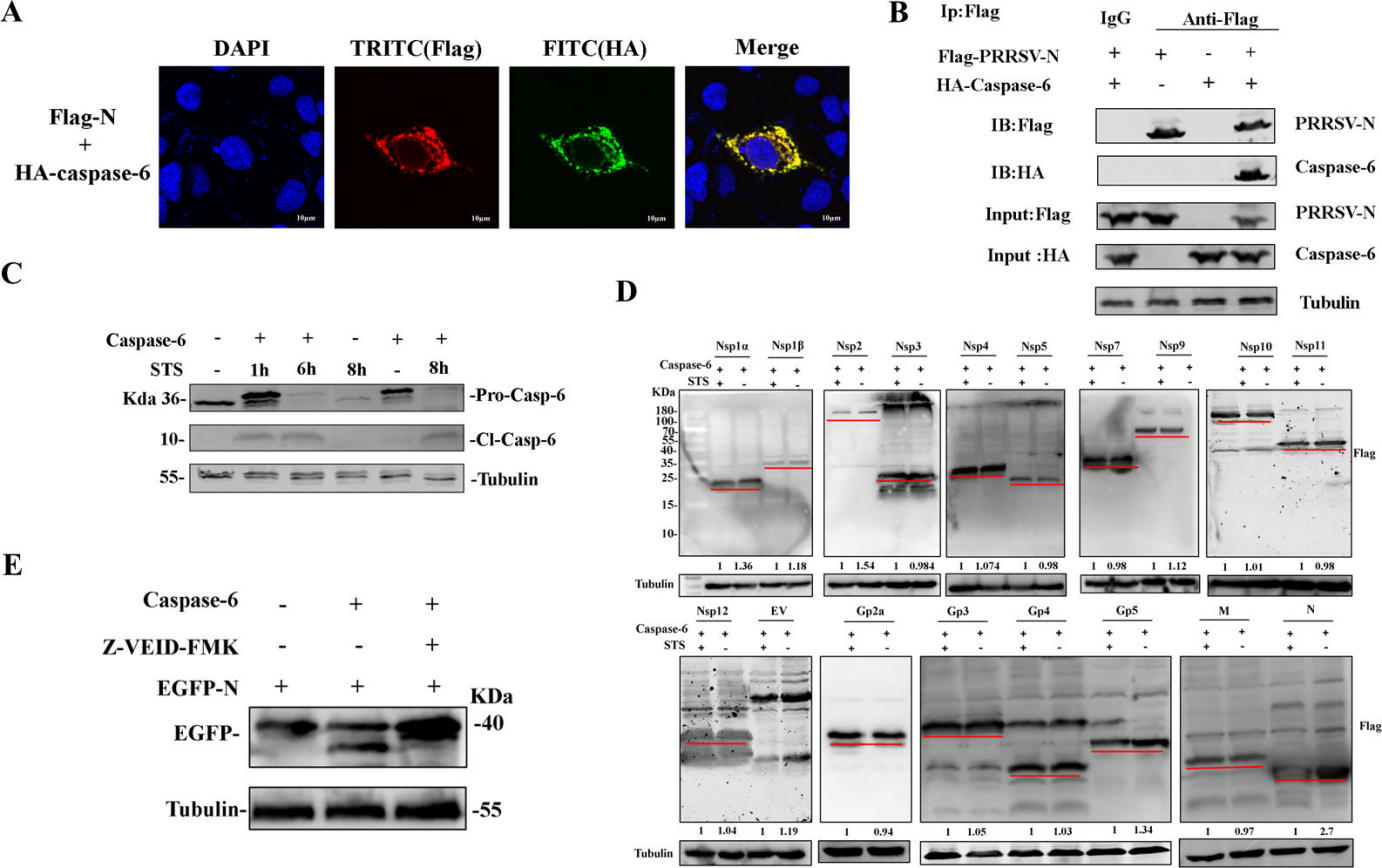
Caspase-6 cleaves PRRSV N protein. (**A**) Confocal immunofluorescence analysis showing the co-localization of PRRSV N protein and caspase-6 in Marc-145 cells co-transfected with Flag-tagged PRRSV N and HA-tagged caspase-6. N protein and caspase-6 were detected using anti-Flag and anti-HA antibodies, respectively. Nuclei were counterstained with DAPI. Scale bars, 10 μm. (**B**) Co-immunoprecipitation analysis of the interaction between caspase-6 and PRRSV N protein. HEK-293T cells were co-transfected with Flag-tagged PRRSV N and HA-tagged caspase-6. Cell lysates were immunoprecipitated with anti-Flag antibody, followed by immunoblotting with anti-HA antibody to detect caspase-6. (**C**) Western blot analysis of caspase-6 activation. HEK-293T cells expressing caspase-6 were treated with staurosporine (STS) to induce apoptosis, and both pro-caspase-6 and cleaved caspase-6 were detected using an anti-caspase-6 antibody. (**D**) Screening of PRRSV structural and nonstructural proteins for caspase-6-mediated cleavage. HEK-293T cells were co-transfected with caspase-6 and the indicated PRRSV viral proteins, and protein cleavage was analyzed by western blotting. Red lines indicate the predicted molecular weights of full-length viral proteins based on amino acid length. Specific cleavage was observed only for the PRRSV N protein. Band intensities were quantified by densitometry using ImageJ, and values shown below each lane represent the relative intensity of the full-length protein normalized to tubulin and further normalized to the corresponding control (set as 1). (**E**) Detection of caspase-6-mediated cleavage of the PRRSV N protein and its inhibition by a specific caspase-6 inhibitor. HEK293T cells were co-transfected with caspase-6 and an EGFP-tagged PRRSV N protein (EGFP-N), in which EGFP was fused to the N terminus to facilitate detection of cleavage fragments. Cells were treated in the presence or absence of caspase-6 overexpression and/or the specific caspase-6 inhibitor Z-VEID-FMK. At 24 h post-transfection, cell lysates were collected and analyzed by western blotting to detect EGFP-N and its cleavage products.

To elucidate the function of caspase-6 in PRRSV replication, caspase-6 was co-expressed with various PRRSV proteins. Confocal microscopy revealed that Nsp9, Nsp10, and the N protein co-localized with caspase-6 in the cytoplasm, appearing as yellow puncta (Fig. 2A; Fig. S1). Further screening via co-immunoprecipitation (Co-IP) confirmed an interaction between the PRRSV N protein and caspase-6 (Fig. 2B; Fig. S2). Caspase-6 is activated by cleavage upon apoptosis induction but can also undergo auto-activation (Fig. 2C). Previous studies have shown that caspase-6 can cleave the N proteins of SARS-CoV-1, TGEV, and PEDV. Caspase-6 was co-expressed with various PRRSV components to evaluate its ability to cleave viral proteins (16, 21, 22). Staurosporine (STS) was used to trigger apoptosis, mimicking the apoptotic environment in PRRSV-infected cells. The results showed a decrease in the expression level of the PRRSV N protein when co-expressed with caspase-6, while other viral components were not cleaved (Fig. 2D). This led us to hypothesize that caspase-6 cleaves the PRRSV N protein. Given the relatively small molecular weight of the N protein (~15 kDa), an EGFP tag was fused to the N-terminus of the N protein. Western blot analysis demonstrated that caspase-6 cleaves the N protein. Moreover, the cleavage of N was inhibited in the presence of the specific caspase-6 inhibitor Z-VEID-FMK (Fig. 2E).

### Caspase-6-mediated cleavage of N protein promotes PRRSV replication by antagonizing the IFN signaling pathway

**Fig 3:**
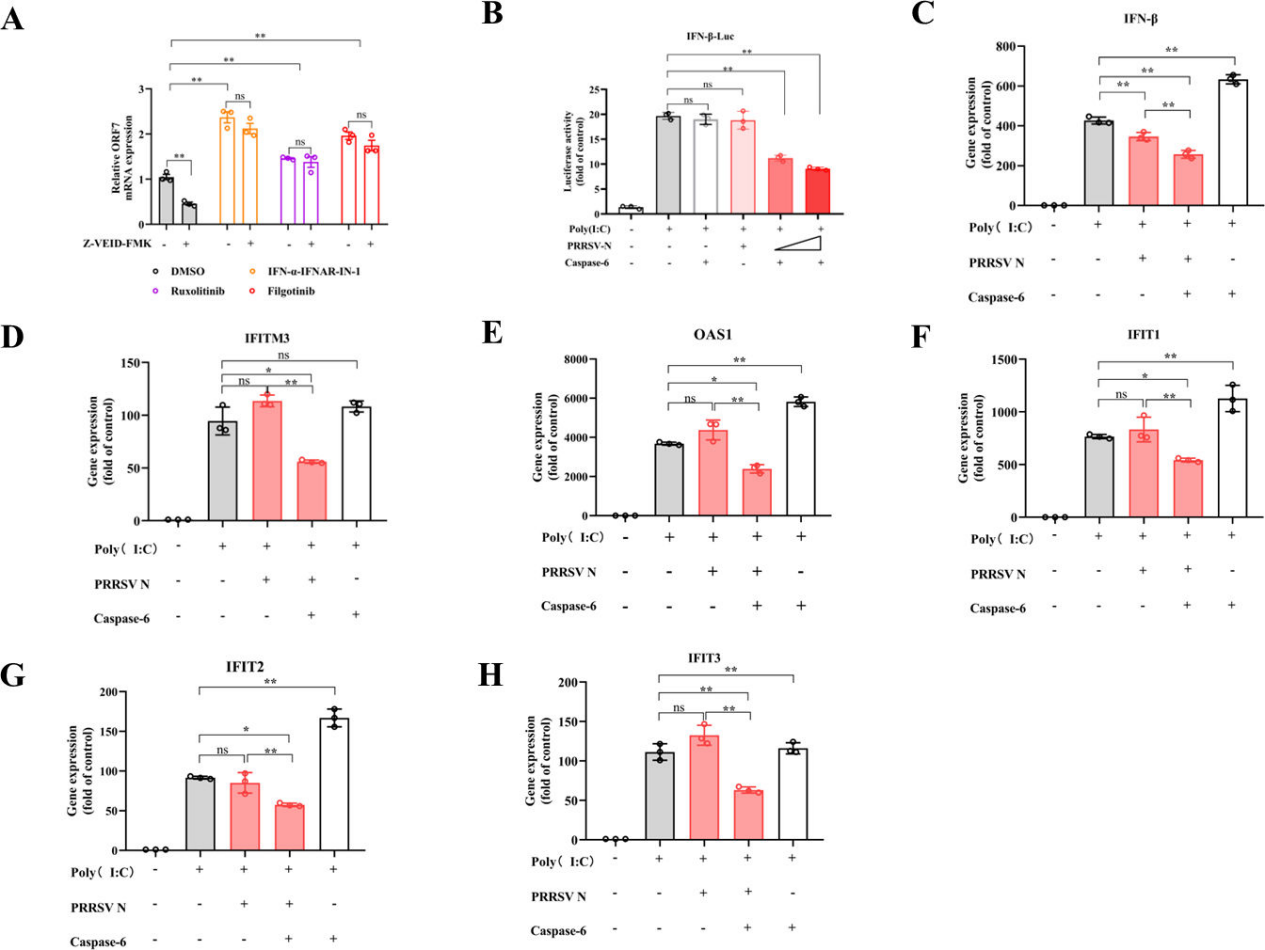
Caspase-6-mediated cleavage of PRRSV N interferes with interferon (IFN) signaling. (**A**) Effect of IFN and JAK pathway inhibition on the antiviral activity of the caspase-6 inhibitor Z-VEID-FMK. Marc-145 cells were pretreated for 2 h with DMSO, IFNAR inhibitor IFN-α–IFNAR-IN-1 (5 μM), JAK1/2 inhibitor ruxolitinib (5 μM), or JAK1 inhibitor filgotinib (5 μM), followed by infection with PRRSV (MOI = 1). Cells were then maintained in medium containing the corresponding inhibitors together with Z-VEID-FMK. At 24 h post-infection (hpi), total RNA was extracted and PRRSV N gene copy numbers were quantified by RT–qPCR and normalized to GAPDH (*n* = 3). (**B**) IFN-β promoter-driven luciferase reporter assays. HEK293T cells were co-transfected with an IFN-β firefly luciferase reporter plasmid, a Renilla luciferase control plasmid, and expression vectors for PRRSV N and caspase-6, in the presence or absence of poly(I:C). At 24 hpi, luciferase activities were measured using a dual-luciferase reporter assay system. Firefly luciferase activity was normalized to Renilla luciferase activity (*n* = 3). (**C–H**) Effect of caspase-6-mediated N cleavage on IFN signaling at the transcriptional level. HEK293T cells were transfected with the IFN-β luciferase reporter plasmid together with PRRSV N and caspase-6 expression constructs, with or without poly(I:C) stimulation. At 24 hpi, total RNA was extracted, and the mRNA levels of IFN-β, IFIT1, IFIT2, IFIT3, IFITM3, and OAS1 were determined by RT–qPCR, normalized to GAPDH, and expressed as fold changes relative to the control group (*n* = 3). Data are presented as mean ± SD from three independent experiments. Statistical significance is indicated as follows: *P < 0.05, **P < 0.01; ns, not significant (*P* ≥ 0.05).

Next, the role of caspase-6-mediated N protein cleavage in regulating PRRSV replication was investigated. PRRSV can suppress the expression of type I IFNs (IFN-α and IFN-β) and IFN-γ, as well as the innate immune signaling pathway (23). To determine whether the cleavage of PRRSV N by caspase-6 affects the IFN pathway, Marc-145 cells infected with PRRSV were treated with Z-VEID-FMK in the presence of DMSO, Filgotinib (a JAK inhibitor), Ruxolitinib (a JAK inhibitor), or IFNα-IFNAR-IN-1 (an IFNAR inhibitor). The antiviral effect of Z-VEID-FMK was attenuated when the IFN pathway was downregulated by JAK or IFNAR inhibition (Fig. 3A). These results suggested that caspase-6-mediated N cleavage may influence PRRSV replication by modulating IFN signaling.

To test this hypothesis, we conducted an IFN-β promoter-driven reporter assay to evaluate the role of caspase-6 and PRRSV N in regulating the IFN response. Notably, co-expression of PRRSV N and caspase-6 inhibited the activation of the IFN-β reporter in a dose-dependent manner (Fig. 3B). In addition to IFN-β reporter activity, the co-expression of caspase-6 and PRRSV N also reduced the mRNA expression of IFN-β and representative IFN-stimulated genes, including *IFIT1*, *IFIT2*, *IFIT3*, *IFITM3*, *TRIM22*, and *OAS1* (Fig. 3C through H).

### Aspartate 94 of PRRSV N protein is the determinant for its IFN-antagonistic function

**Fig 4:**
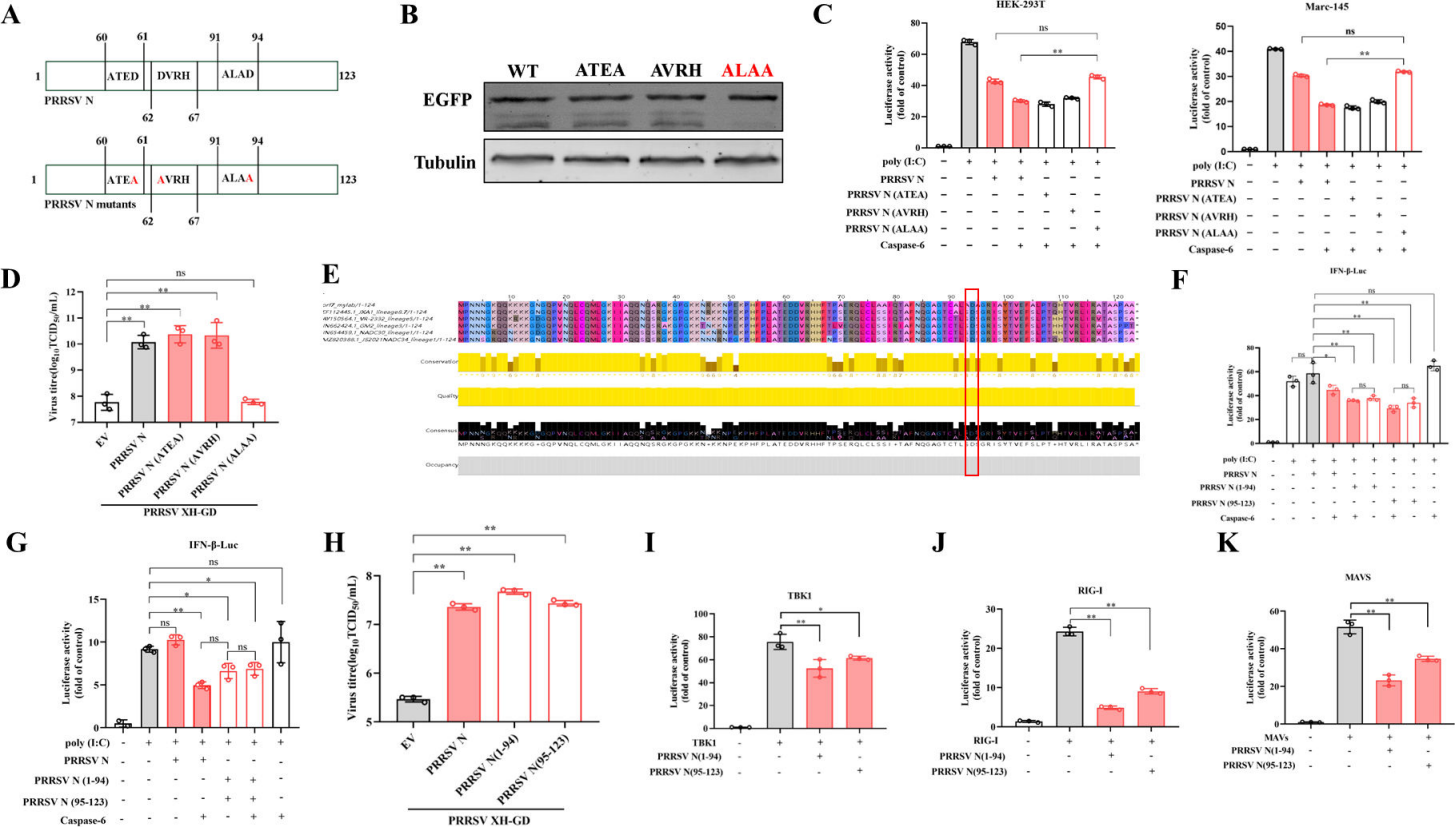
Caspase-6 cleaves N at Aspartate 94. (**A**) Schematic representation of PRRSV N protein and the caspase-6 cleavage site mutants. Based on predicted caspase-6 recognition motifs, aspartate residues at positions 61, 62, and 94 were individually mutated to alanine to generate the D61A, D62A, and D94A mutants. (**B**) Western blot analysis of caspase-6-mediated cleavage of PRRSV N and its mutants. HEK293T cells were co-transfected with caspase-6 and wild-type or mutant N constructs, and protein cleavage was analyzed by immunoblotting. Cleavage was abolished in the D94A mutant. (**C**) IFN-β promoter-driven luciferase reporter assays in HEK-293T and Marc-145 cells. Cells were co-transfected with IFN-β luciferase reporter plasmid together with caspase-6 and PRRSV N or N mutants, with or without poly(I:C) stimulation. Luciferase activities were measured at 24 h post-transfection (hpi) and normalized to Renilla luciferase activity (*n* = 3). (**D**) Viral titers in Marc-145 cells following overexpression of PRRSV N or N mutants and subsequent infection with PRRSV. Viral titers were determined at 24 hpi. (**E**) Sequence alignment showing conservation of the caspase-6 cleavage site at aspartate 94 among representative PRRSV strains. (**F, G**) IFN-β luciferase reporter assays assessing the activity of caspase-6-cleaved N protein fragments. HEK-293T cells were transfected with IFN-β reporter plasmid together with caspase-6 and full-length N, N (1–94), or N (95–123), with or without poly(I:C). Luciferase activity was measured at 24 hpi (*n* = 3). (**H**) Viral titers in Marc-145 cells following overexpression of N (1–94) or N (95–123) fragments and PRRSV infection. (I–K) Mapping the point of action of N cleavage fragments within the IFN signaling pathway. HEK293T cells were co-transfected with IFN-β luciferase reporter plasmid and expression vectors for TBK1 (**I**), RIG-I (**J**), or MAVS (**K**), together with PRRSV N (1–94) or N (95–123). Luciferase activity was measured at 24 hpi, indicating that the N cleavage fragments act downstream of RIG-I, MAVS, and TBK1. Data are presented as mean ± SD from three independent experiments. Statistical significance is indicated as *P < 0.05, **P < 0.01; ns, not significant (*P* ≥ 0.05).

To further elucidate how caspase-6-mediated N cleavage regulates the IFN response, we analyzed potential caspase-6 cleavage sites on the PRRSV N protein based on known caspase-6 substrate specificity and the size of the cleavage fragments (24). We designed and constructed corresponding N mutants, designated D61A (ATED to ATEA), D62A (DVRH to AVRH), and D94A (ALAD to ALAA) (Fig. 4A). A western blot-based cleavage assay revealed that caspase-6-mediated cleavage was abolished in the D94A mutant, indicating that caspase-6 cleaves PRRSV N at the ALAD motif (Fig. 4B). In the IFN-β reporter assay, the IFN-antagonistic effect mediated by caspase-6 and N was attenuated when aspartate 94 was mutated to alanine (D94A) (Fig. 4C). Furthermore, when Marc-145 cells were transfected to overexpress the various mutants and subsequently infected with PRRSV, the D94A mutant no longer promoted an increase in viral titer (Fig. 4D). This cleavage site is conserved across different PRRSV strains (Fig. 4E). These findings demonstrate that caspase-6-mediated cleavage of PRRSV N is crucial for its IFN-antagonistic function.

Next, we constructed two fragments, N (1–94) and N (95–123), to mimic the N protein cleavage products. In the IFN-β reporter assay, both N cleavage products independently restricted IFN-β reporter activity, but this effect was no longer modulated by caspase-6 (Fig. 4F and G). These fragments could also directly promote PRRSV replication (Fig. 4H). To dissect how the PRRSV N fragments regulate IFN signaling, we assessed their point of action using a luciferase reporter assay with RIG-I, MAVS, and TBK1 as activators. The results showed that the PRRSV N fragments effectively reduced IFN-β luciferase reporter activity upon activation by RIG-I, MAVS, or TBK1, indicating that the N fragments act downstream of these signaling molecules (Fig. 4I through K).

### Caspase-6 cleavage of N enhances its interaction with IRF3

**Fig 5:**
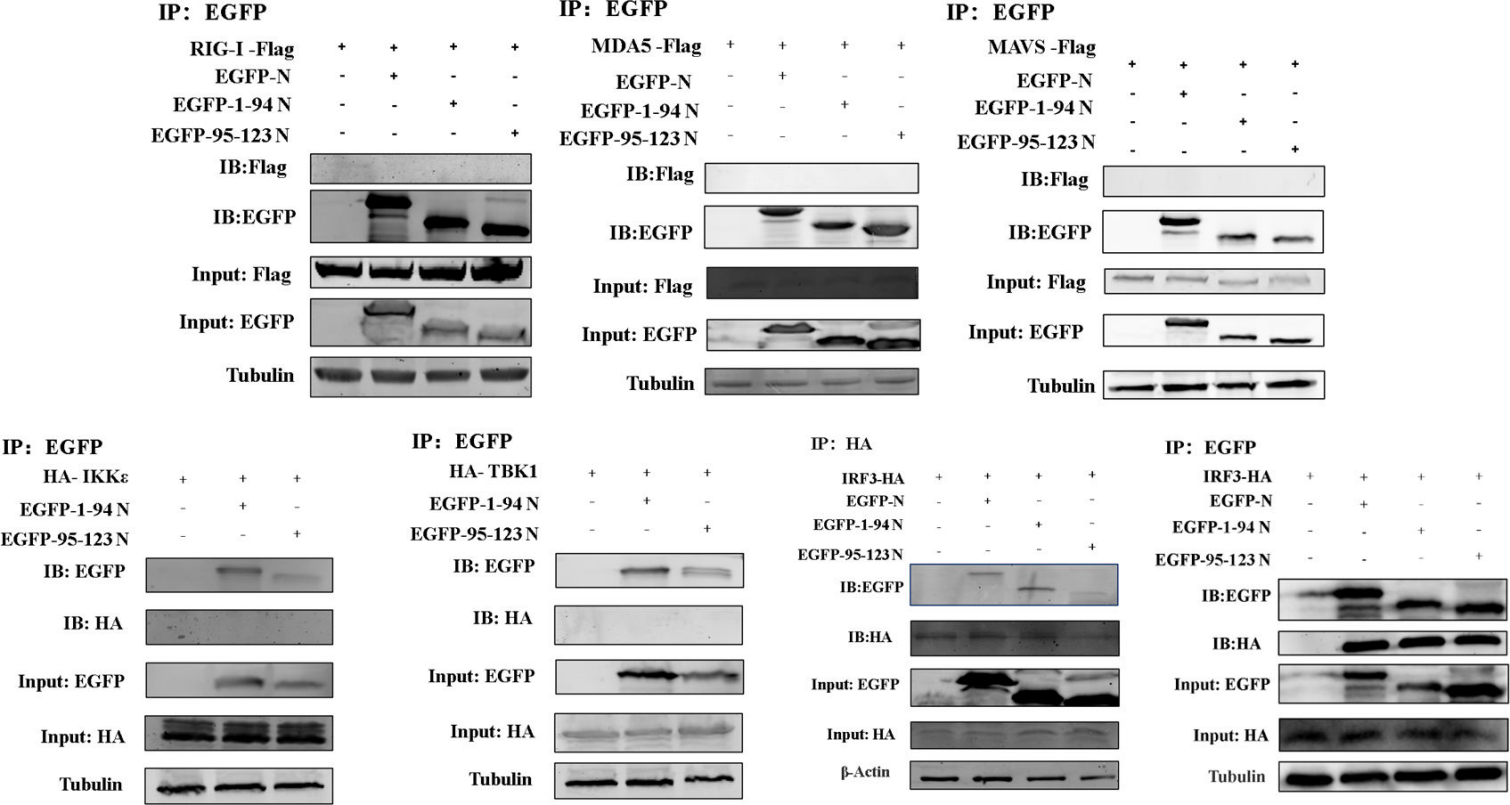
PRRSV N protein cleavage fragments interact with IRF3. Co-immunoprecipitation analysis of the interaction between PRRSV N protein and its caspase-6-generated cleavage fragments with components of the IFN signaling pathway. HEK293T cells were co-transfected with expression constructs encoding full-length PRRSV N, N (1–94), or N (95–123) together with IRF3. Cell lysates were immunoprecipitated using antibodies against the tagged N constructs, followed by immunoblotting with anti-IRF3 antibody. The results demonstrate that both full-length N protein and its cleavage fragments interact with IRF3; no interaction was detected with other upstream IFN signaling components.

**Fig 6:**
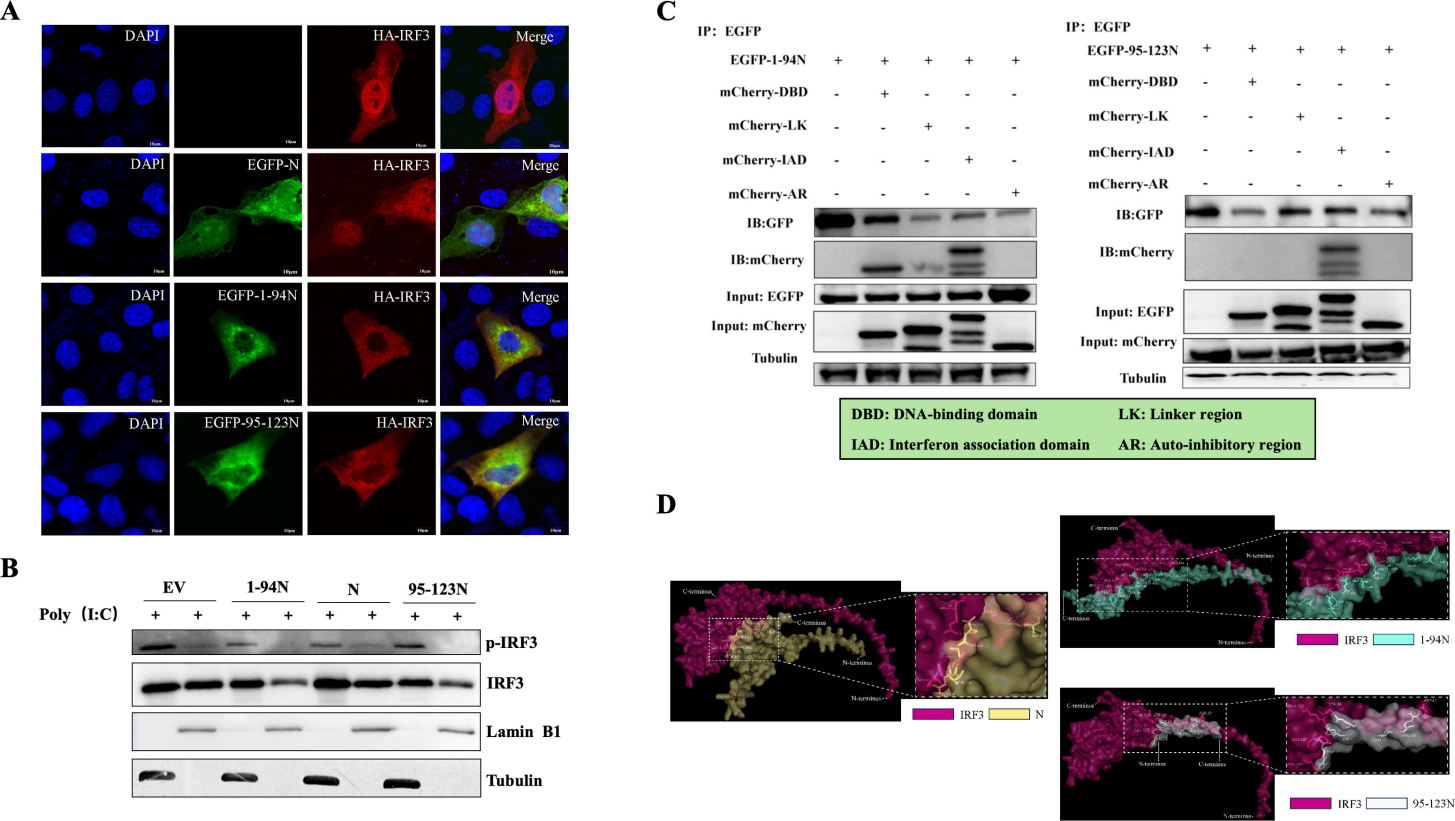
Caspase-6-cleaved PRRSV N fragments co-localize with IRF3 and inhibit its nuclear translocation. (**A**) Confocal immunofluorescence analysis of the subcellular localization of IRF3 in the presence of PRRSV N protein or its cleavage fragments. Marc-145 cells were co-transfected with HA-tagged IRF3 and EGFP-tagged PRRSV N, N (1–94), or N (95–123) expression constructs, together with poly(I:C) to activate IRF3 signaling. At 24 h post-transfection (hpi), cells were fixed and stained with anti-HA antibody to detect IRF3. Nuclei were counterstained with DAPI. Scale bars, 10 μm. (**B**) Nuclear and cytoplasmic fractionation analysis of IRF3 localization. Marc-145 cells were co-transfected as described for panel A, and nuclear and cytoplasmic extracts were prepared using a nucleocytoplasmic separation kit at 24 hpi. IRF3 distribution was analyzed by western blotting, demonstrating that PRRSV N cleavage fragments inhibit poly(I:C)-induced nuclear translocation of IRF3. (**C**) Mapping of the interaction domains between PRRSV N protein cleavage fragments and IRF3. Schematic representation showing that N (1–94) interacts with both the DNA-binding domain (DBD) and IRF-association domain (IAD) of IRF3, whereas N (95–123) interacts primarily with the DBD. (**D**) *In silico* three-dimensional structural modeling of the interaction between PRRSV N cleavage fragments and IRF3. Structural simulations were generated using PyMOL to illustrate potential interaction interfaces and to provide a structural rationale for the enhanced binding of cleaved N fragments to IRF3.

To elucidate the influence of PRRSV N protein cleavage products on the IFN signaling pathway, the full-length N protein and its fragments N (1–94) and N (95–123) were analyzed for their interactions with pathway components. All three forms of N were found to bind to IRF3, suggesting that they modulate the IFN response by interfering with IRF3 function. No interaction was detected with other elements of the IFN pathway (Fig. 5). In Marc-145 cells stimulated with the TLR3 agonist poly(I:C), IRF3 translocated into the nucleus regardless of PRRSV N expression (Fig. 6A). Further analysis using nuclear and cytoplasmic fractionation demonstrated that the N (1–94) and N (95–123) fragments co-localized with IRF3 and blocked its nuclear translocation (Fig. 6B).

Because IRF3 nuclear import depends on karyopherins KPNA1, KPNA2, KPNA3, and KPNA4 (25). Additional assays were conducted to exclude the possibility that N cleavage fragments disrupt this transport process. Co-expression of N and its cleavage fragments with KPNA1, KPNA2, KPNA3, and KPNA4 revealed no interaction in Co-IP assays, indicating that the inhibitory effect of the N fragments on IRF3 function is not mediated through karyopherin regulation (Fig. S3).

To identify the domains of IRF3 that interact with the PRRSV N cleavage fragments, western blotting was conducted to map their binding regions. The results showed that the PRRSV N (1–94) fragment specifically interacted with both mCherry-DBD and mCherry-IAD, whereas the N (95–123) fragment only interacted with mCherry-DBD (Fig. 6C). Subsequently, a 3D interaction model was generated using PyMOL combined with PDBePISA analysis. The structural analysis indicated that, compared with the full-length, uncleaved N (1–123) protein, the caspase-6-cleaved N (1–94) and N (95–123) fragments exposed more potential interaction sites (Fig. 6D). Among these, Ser251, Ser101, and Arg105 are key residues that directly regulate nuclear transport, while Glu53, Thr69, Glu55, Glu148, Gln64, Val65, Arg63, and Gly99 indirectly regulate nuclear transport and function by affecting phosphorylation, dimerization, DNA binding, and conformational changes. Collectively, the cleaved N protein antagonizes IRF3-mediated antiviral responses through synergistic, multi-site interactions. These results suggest that the cleavage of the N protein may enhance its ability to interact with IRF3, thereby interfering with IRF3-mediated antiviral signaling.

### PRRSV-D94A mutant exhibits attenuated virulence and enhanced immunogenicity *in vivo*

**Fig 7:**
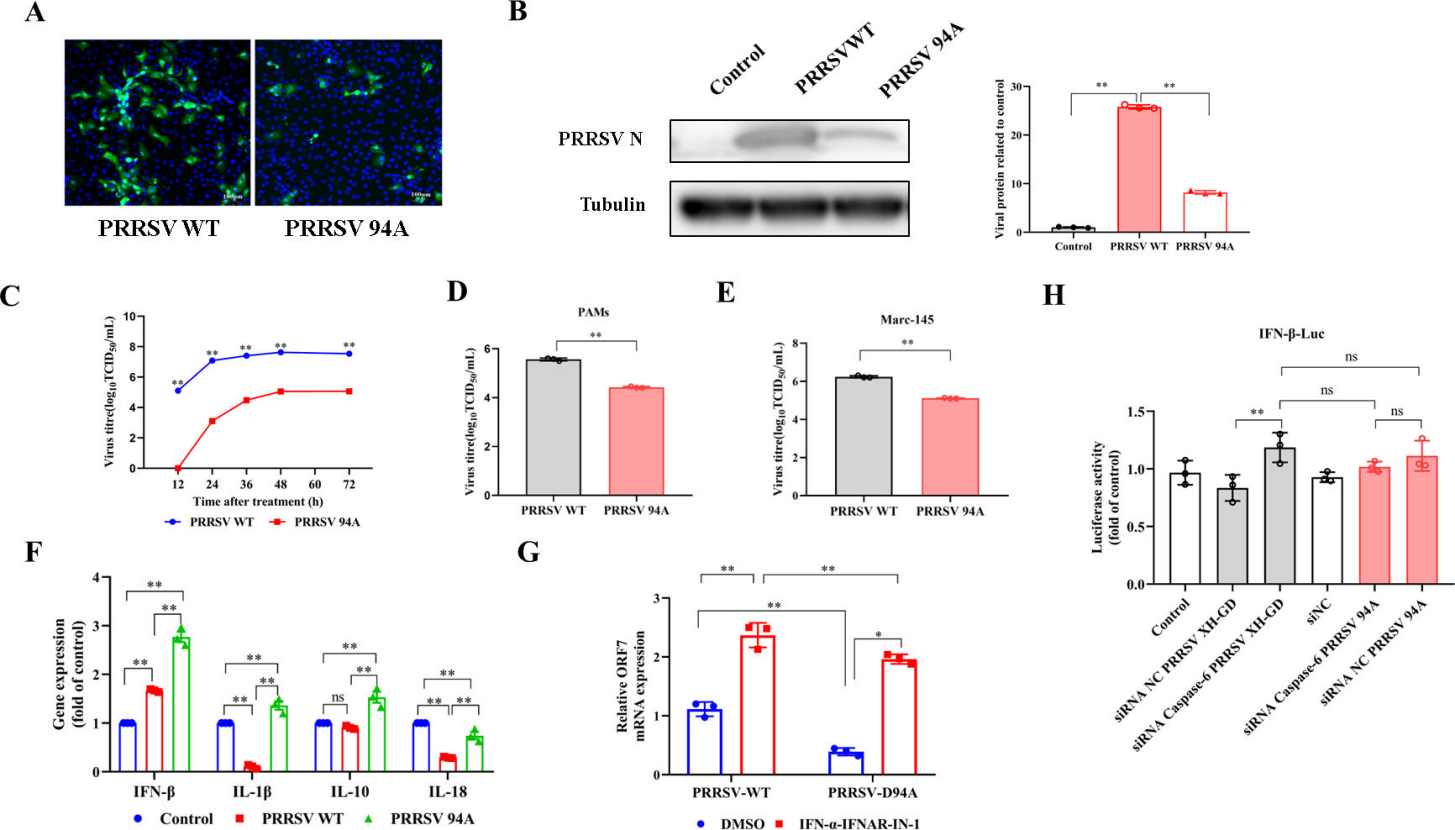
Attenuated *in vitro* replication capacity of the mutant strain. (**A**) Immunofluorescence analysis of viral replication in Marc-145 cells infected with wild-type PRRSV (PRRSV-WT) or the D94A mutant strain. Cells were infected at a multiplicity of infection (MOI) of 1 and fixed at 24 h post-infection (hpi). PRRSV N protein was detected by immunofluorescence staining. Nuclei were counterstained with DAPI. (**B**) Western blot analysis of PRRSV N protein expression in Marc-145 cells infected with PRRSV-WT or PRRSV-D94A at MOI = 1. Cell lysates were collected at 24 hpi, and tubulin was used as a loading control. (**C**) One-step growth curves of PRRSV-WT and PRRSV-D94A in Marc-145 cells. Cells were infected at MOI = 1, and viral titers in the supernatants were determined at the indicated time points by TCID₅₀ assay. (**D, E**) Viral titers of PRRSV-WT and PRRSV-D94A in porcine alveolar macrophages (PAMs) (**D**) and Marc-145 cells (**E**). Cells were infected at MOI = 1, and viral titers were measured at 24 hpi by TCID₅₀ assay. (**F**) Host innate immune responses induced by PRRSV-WT and PRRSV-D94A infection. Marc-145 cells were infected at MOI = 1, and the mRNA levels of IFN-β, IL-1β, IL-10, and IL-18 were quantified by RT–qPCR at 24 hpi (*n* = 3). (**G**) Effect of interferon signaling blockade on viral replication. Marc-145 cells infected with PRRSV-WT or PRRSV-D94A were treated with IFN-α–IFNAR-IN-1 or DMSO as control. Viral replication was assessed by measuring ORF7 mRNA levels via RT–qPCR at 24 hpi (*n* = 3). (**H**) IFN-β promoter activity in Marc-145 cells infected with PRRSV-WT or PRRSV-D94A in the presence or absence of caspase-6 knockdown. Cells were transfected with an IFN-β luciferase reporter plasmid together with control or caspase-6-specific siRNA, followed by viral infection. Luciferase activities were measured at 24 hpi and normalized to Renilla luciferase activity (*n* = 3). Data are presented as mean ± SD from three independent experiments. Statistical significance is indicated as *P < 0.05, **P < 0.01; ns, not significant (*P* ≥ 0.05).

To further investigate the *in vitro* roles of caspase-6-mediated N protein cleavage, a PRRSV-D94A mutant virus was constructed and rescued using a reverse genetics system. The viral replication capacity and N protein fluorescence intensity of PRRSV-D94A were lower than those of the wild-type strain (PRRSV-WT) (Fig. 7A and B). Furthermore, a one-step growth curve analysis revealed that the viral titers of the mutant strain were significantly lower than those of the PRRSV-WT strain across all time points (*P* < 0.05) (Fig. 7C); endpoint titration assays in both porcine alveolar macrophages (PAMs) and Marc-145 cells further confirmed a sustained decrease in viral yield (Fig. 7D and E). Given the attenuated replication of the PRRSV-D94A mutant, we assessed whether this phenotype was associated with altered host innate immune responses. Compared with PRRSV-WT infection, PRRSV-D94A induced significantly higher mRNA levels of IFN-β, IL-1β, IL-10, and IL-18 in infected cells (*P* < 0.05), indicating a reduced capacity of the mutant virus to antagonize innate immune signaling (Fig. 7F). To directly determine whether the replication defect of PRRSV-D94A is dependent on interferon signaling, type I IFN signaling was pharmacologically blocked using the IFNAR inhibitor IFN-α–IFNAR-IN-1. Under IFNAR blockade, viral replication of both PRRSV-WT and PRRSV-D94A was significantly enhanced, as measured by ORF7 mRNA levels. Importantly, replication of the PRRSV-D94A mutant was markedly restored, although not fully to WT levels, indicating that the attenuated replication of PRRSV-D94A is largely, but not exclusively, IFN-dependent (Fig. 7G). Furthermore, caspase-6 knockdown markedly impaired the ability of PRRSV-WT to suppress IFN-β promoter activity, whereas IFN antagonism by the PRRSV-D94A mutant was no longer affected by caspase-6 depletion (Fig. 7H). These results indicate that the D94A mutation functionally uncouples viral immune antagonism from caspase-6-mediated regulation, thereby contributing to the attenuated replication phenotype of the mutant virus.

**Fig 8:**
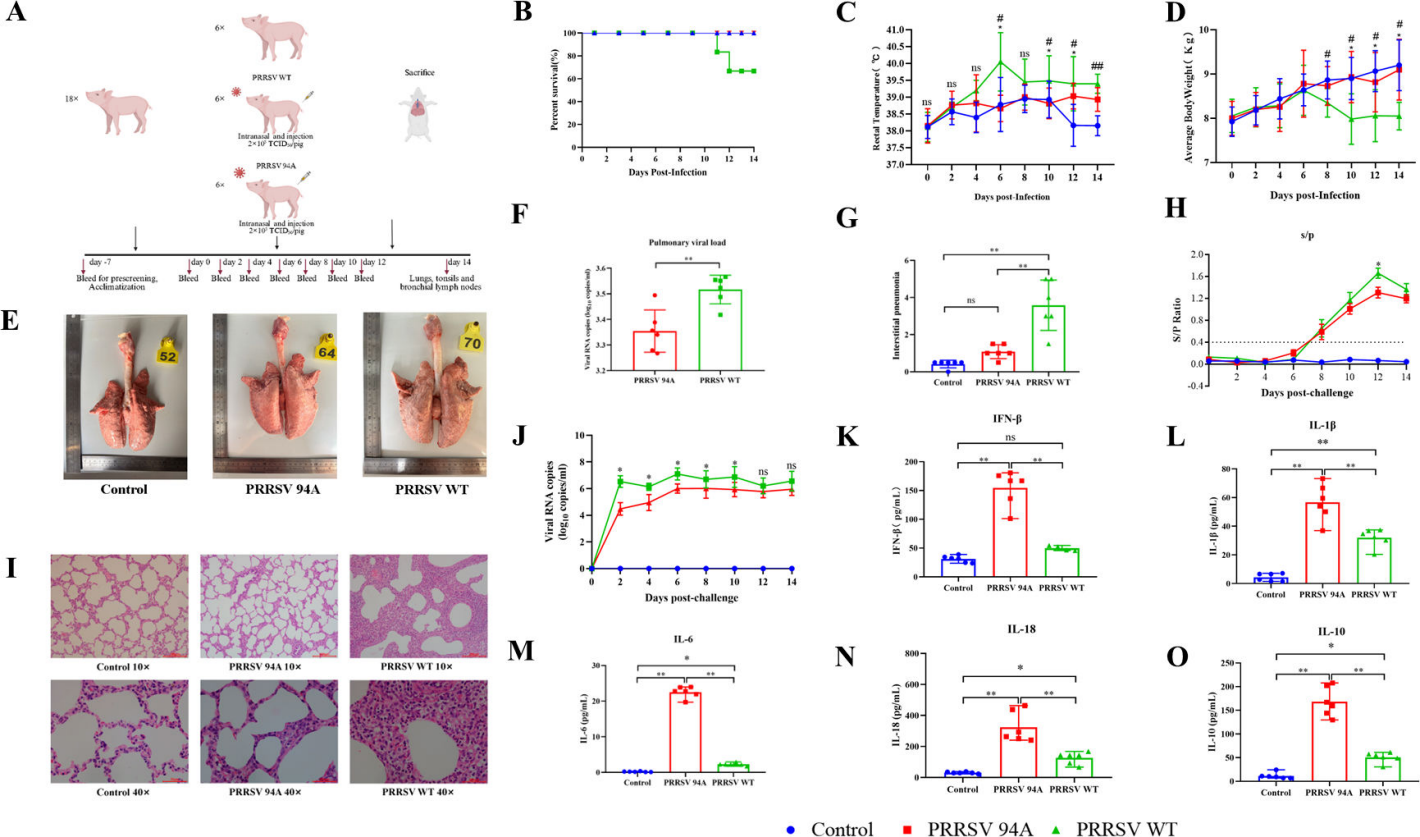
Reduced *in vivo* replication capacity and pathogenicity of the mutant strain. (**A**) Schematic overview of the animal experiment design. Four-week-old piglets were randomly divided into three groups (*n* = 6 per group) and challenged intranasally and intramuscularly with wild-type PRRSV (WT) or the D94A mutant strain at a dose of 2 × 10⁵ TCID₅₀ per pig. A mock-infected group served as a control. (**B**) Survival curves of piglets infected with WT or D94A mutant PRRSV over the observation period. (**C**) Daily rectal temperature changes in piglets following infection. (**D**) Daily body weight changes in piglets following infection. (**E**) Gross pathological examination of lung tissues collected from euthanized piglets at the experimental endpoint. Representative images are shown. (**F**) Viral RNA loads in lung tissues of piglets infected with WT or D94A mutant PRRSV. Total RNA was extracted from lung tissues, and viral RNA levels were quantified by RT–qPCR. (**G**) Histopathological scoring of interstitial pneumonia in lung tissues. Scoring was performed in a blinded manner based on hematoxylin and eosin (H&E)-stained sections. (**H**) Serological analysis of PRRSV-specific antibodies in piglet sera. Antibody levels are expressed as S/P ratios measured by ELISA, with an S/P ratio ≥ 0.4 considered positive. (**I**) Representative histological images of lung sections from infected piglets stained with H&E. Images were acquired at 10× and 40× magnification. (**J**) Viral RNA loads in serum samples collected from piglets at the indicated days post-infection, quantified by RT–qPCR. (**K–O**) Serum cytokine levels in piglets infected with WT or D94A mutant PRRSV. Concentrations of IFN-β (**K**), IL-1β (**L**), IL-6 (**M**), IL-18 (**N**), and IL-10 (**O**) were measured by ELISA at the indicated time points. Data are presented as mean ± SD. Statistical significance is indicated as *P < 0.05, **P < 0.01; ns, not significant (*P* ≥ 0.05).

In the *in vivo* challenge experiment, piglets infected with PRRSV-WT exhibited persistent high fever and a mortality rate of 33.3%, whereas no deaths were recorded in the PRRSV-D94A group (Fig. 8B). Body temperature fluctuations were also significantly less pronounced in the D94A group than in the WT group (Fig. 8C). Weight monitoring showed that animals in the PRRSV-WT group lost weight after day 6 post-infection, while the PRRSV-D94A group maintained normal growth (*P* < 0.05) (Fig. 8D). Pathological examination revealed extensive lung consolidation and severe interstitial pneumonia in PRRSV-WT-infected pigs, whereas the PRRSV-D94A group showed only focal, mild lesions (Fig. 8E). Consistently, quantitative RT-PCR analysis demonstrated that the pulmonary viral load in the PRRSV-D94A group was significantly lower than that in the PRRSV-WT group (*P* < 0.01) (Fig. 8F). HE staining further confirmed that PRRSV-D94A infection resulted in reduced inflammatory cell infiltration and well-preserved alveolar structures in lung tissue (Fig. 8I). The pathological score for interstitial pneumonia was significantly lower in the PRRSV-D94A group than in the PRRSV-WT group (*P* < 0.01), consistent with the body temperature and weight change data (Fig. 8G).

Serum viremia analysis showed that the viral load in the PRRSV-D94A group was consistently lower than that in the PRRSV-WT group from day 2 to day 10 post-infection (*P* < 0.05), with a markedly lower peak (Fig. 8H). Despite the lower viremia, ELISA results indicated that both groups produced PRRSV N-specific antibodies after day 8, although the antibody level in the PRRSV-D94A group was slightly lower than that in the WT group on day 12 (*P* < 0.05) (Fig. 8J). Cytokine analysis revealed that the IFN-β levels induced by PRRSV-D94A were significantly higher than in the WT group (*P* < 0.01) (Fig. 8K). Pro-inflammatory cytokines IL-1β, IL-6, and IL-18 were concurrently upregulated (*P* < 0.05) (Fig. 8L through N), along with an increase in the anti-inflammatory cytokine IL-10 (Fig. 8O), suggesting that the mutant virus induced a stronger yet immunomodulated inflammatory response. Sanger sequencing of samples from the lung, tonsil, lymph nodes, and serum confirmed that the D94A mutation did not revert or acquire new mutations *in vivo* (Table S3; Fig. S5).

In summary, the PRRSV-D94A mutant demonstrated attenuated replication and pathogenicity both *in vitro* and *in vivo*, and it induced a more potent IFN and inflammatory cytokine response. With its good genetic stability, it shows significant potential as a live attenuated vaccine candidate.

## DISCUSSION

**Fig 9:**
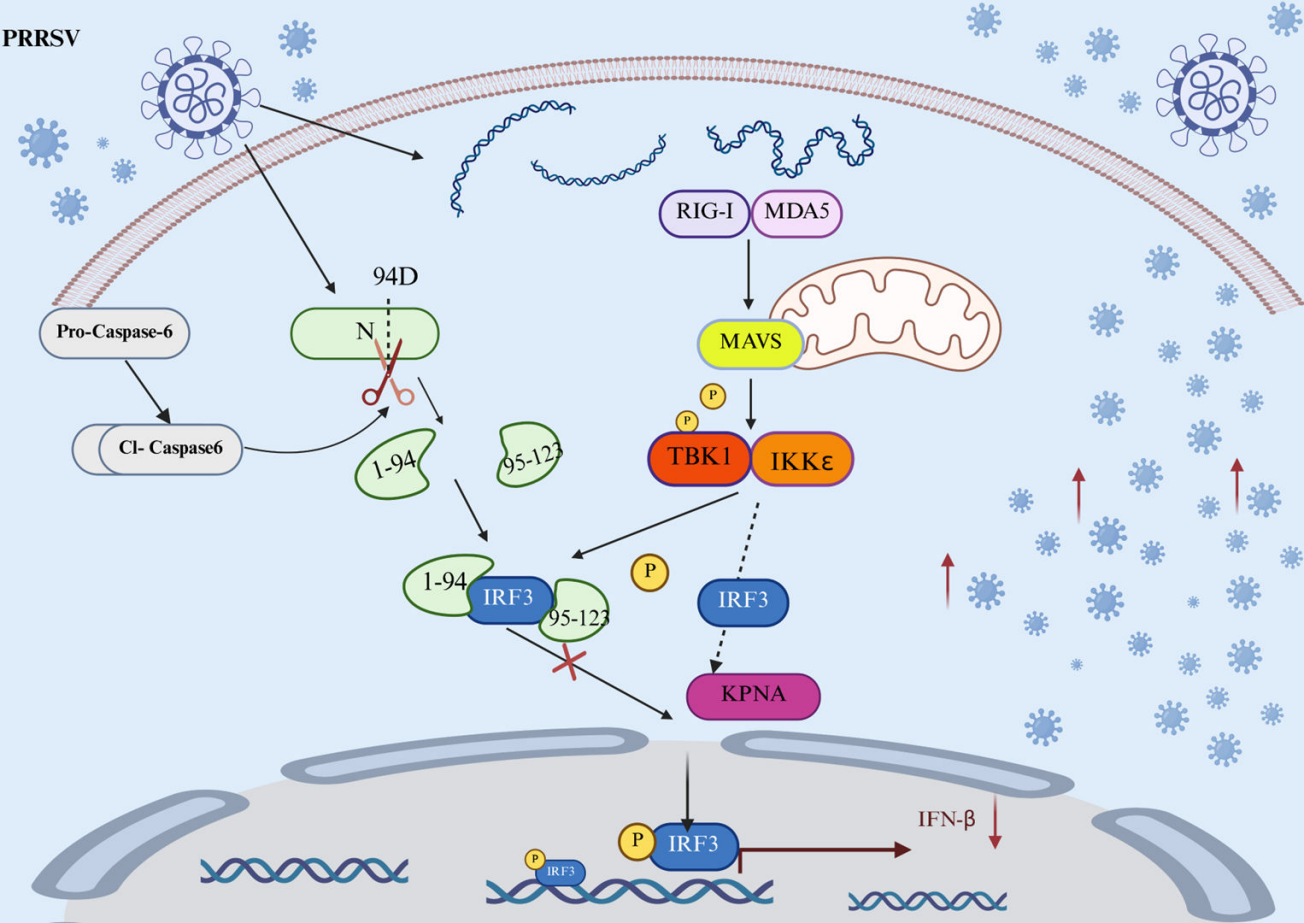
Model illustrating how PRRSV exploits caspase-6 to facilitate its replication. PRRSV employs caspase-6 to cleave its N protein at aspartic acid residue 94 (D94), generating an N protein fragment capable of interacting with IRF3. This interaction attenuates the activation of IFN signaling, thus enhancing viral replication.

The dynamic interplay between viruses and the host cell’s programmed cell death pathways represents a critical juncture in the viral life cycle. As a conserved host defense mechanism, apoptosis is often manipulated by viruses to achieve a dynamic equilibrium between viral replication and host survival (26, 27). The regulation of host cell apoptosis during PRRSV infection has been widely reported, with the virus selectively inducing or inhibiting apoptotic signals depending on the infection stage and host cell type to optimize its replication efficiency (28). This study, for the first time, systematically elucidates a molecular mechanism, whereby PRRSV specifically recruits the host effector enzyme caspase-6 to mediate the cleavage of its N protein at a specific site. This action leads to the suppression of the type I IFN signaling pathway, thereby promoting viral replication (Fig. 9). This finding is further corroborated by previous reports. Zhang et al. (2016) demonstrated that the PRRSV N protein suppresses IFN-β production by inhibiting IRF3 activation and nuclear translocation in immortalized porcine alveolar macrophages (29). Building upon this, the current study revealed that the immunosuppressive function of the N protein is closely regulated through site-specific cleavage mediated by host caspase-6, thereby providing a mechanistic link between viral protein processing and innate immune evasion. This insight underscores the multi-functional role of the N protein as a pivotal node in orchestrating both viral replication and immune suppression.

In this context, notably, a conceptually related mechanism has been reported for SARS-CoV-2—another member of the order *Nidovirales*. Chu et al. (30) demonstrated that coronaviruses exploit a host cysteine–aspartic protease, caspase-6, to promote viral replication through direct cleavage of the viral nucleocapsid protein. Our findings extend this paradigm to arteriviruses by showing that PRRSV similarly hijacks host caspase-6 to cleave its N protein, thereby suppressing IRF3-mediated type I interferon signaling and facilitating viral replication. Notably, despite differences in genome organization, host range, and pathogenic outcomes between coronaviruses and arteriviruses, both viruses converge on caspase-6 as a host factor to modulate nucleocapsid function and innate immune responses. These observations suggest that exploitation of caspase-6 may represent a conserved strategy among nidoviruses to optimize replication through proteolytic remodeling of viral structural proteins.

Caspase-6, a key effector enzyme of the caspase family, has traditionally been associated with neurodegenerative diseases; however, its role in viral infections has not been fully explored (31, 32). Our study reveals that the activation of host caspase-6 is not merely an epiphenomenon of viral infection but plays a pivotal role in the viral replication cycle. In our functional screening using both siRNA-mediated knockdown and selective caspase inhibitors (Fig. 1D through H), caspase-6 displayed a highly consistent phenotype across experimental approaches: both genetic silencing and pharmacological inhibition with Z-VEID-FMK resulted in a significant reduction in PRRSV titers, thereby guiding our decision to prioritize caspase-6 for subsequent mechanistic analyses. In contrast, other caspases exhibited more heterogeneous effects depending on the mode of intervention. For example, treatment with the caspase-1/5 inhibitor Z-WEHD-fmk markedly enhanced viral replication, whereas siRNA-mediated knockdown of caspase-1 alone did not fully recapitulate this phenotype. Consistent with the known functional redundancy among inflammasome-associated effector caspases, combined knockdown of caspase-1 and caspase-5 promoted PRRSV replication to a level comparable to that induced upon Z-WEHD-FMK treatment, indicating that the observed proviral effect reflects collective suppression of the inflammasome-related caspase module rather than a caspase-1-specific proviral role. (Fig. S4) Notably, inhibitors targeting several other caspases, including initiator caspases such as caspase-8 and caspase-9, also resulted in increased or minimal changes in viral replication. Given that these caspases participate in diverse cellular processes beyond linear apoptotic cascades, such as regulation of inflammatory signaling, cell fate decisions, and innate immune responses, their inhibition is likely to attenuate host restriction mechanisms rather than directly modulate viral replication machinery. Together, these findings underscore a functional divergence within the caspase family during PRRSV infection, with caspase-6 uniquely acting as a host factor directly exploited by PRRSV at the molecular level. Of particular note is the complex, dynamic regulation of caspase-6 function we observed: both overexpression and siRNA-mediated knockdown of caspase-6 resulted in significant inhibition of PRRSV replication. This “bidirectional inhibition” is not unique; similar phenomena have been reported in studies of Zika virus and Dengue virus infections, where the effect of a key host protein on viral replication can be reversed at different expression levels (33, 34). This suggests that the viral exploitation of host factors is not a simple linear relationship but depends on precise expression thresholds and the intracellular microenvironment of the host factor. Importantly, this bidirectional effect does not imply opposing roles of caspase-6 at the same mechanistic level, but rather reflects the balance between N protein cleavage–mediated immune suppression and apoptosis-driven premature termination of viral replication. At physiological or moderately activated levels, caspase-6 mediates the specific cleavage of PRRSV N protein at D94, executing the immunosuppressive function required by the virus. However, excessive expression or activity of caspase-6 may accelerate host cell apoptosis, leading to premature termination of the viral replication cycle and ultimately reducing viral yield. Conversely, insufficient or absent caspase-6 fails to achieve the necessary protein modification and immunosuppression, also resulting in decreased replication efficiency. Further investigation revealed a significant interaction between caspase-6 and the PRRSV N and Nsp9 proteins. The interaction between N and Nsp9 is essential for viral RNA synthesis and replication. As Nsp9 is a core component of the replication-transcription complex, caspase-6 may be recruited to the viral replication–transcription complex, where it primarily facilitates immune evasion through site-specific cleavage of the N protein rather than broadly modulating the viral replication machinery (35, 36). Sequence analysis further confirmed that the D94 residue is highly conserved among multiple representative PRRSV strains (e.g., JXA1, VR2332, NADC30, NADC34), highlighting its functional importance in the viral life cycle and its potential as a vaccine target.

This dynamic, bidirectional regulatory mechanism further corroborates the complex and sophisticated interplay between the host and virus on the apoptotic signaling pathway. The virus must strike a delicate balance between promoting and inhibiting host cell apoptosis to optimize its own replication and transmission efficiency. Thus, the homeostatic state of caspase-6 function may represent a critical node in the viral regulation of host cell fate, providing a new conceptual framework and theoretical basis for designing future antiviral drugs based on the modulation of host cell apoptosis.

To further clarify the function of the D94 site in PRRSV infection, this study constructed an uncleavable mutant, PRRSV-D94A, using reverse genetics and evaluated its replication capacity and immunogenicity in cellular and animal models. Both models consistently showed that the replication capacity of the PRRSV-D94A strain was significantly attenuated, which was accompanied by a marked upregulation of host type I IFN and inflammatory cytokine expression. This indicates that the D94A mutation disrupts the virus’s ability to exploit host factors for innate immune evasion, allowing for effective activation of the host immune response and a significant reduction in viral load. The increased IFN levels not only directly inhibit viral replication but also enhance the host’s overall antiviral defense by activating innate immune effector cells such as NK cells and dendritic cells (12, 37, 38). Concurrently, the upregulation of IL-10 may effectively prevent an excessive inflammatory response (i.e., a “cytokine storm”), achieving a dynamic balance between immune activation and suppression (39, 40). In the animal model, pigs infected with PRRSV-D94A exhibited mild clinical signs, reduced viremia, significantly less lung damage, and enhanced immunogenicity, suggesting the mutant’s strong potential as a live attenuated vaccine strain.

A growing body of research indicates that the hijacking of the host caspase pathway is not unique to PRRSV. Coronaviruses (e.g., SARS-CoV-2) and influenza viruses can also exploit host caspase pathways to evade immune surveillance (17, 41, 42). This study is the first to place PRRSV within the family of RNA viruses that achieve immune evasion through host caspase-6, thereby expanding our understanding of virus–host interaction mechanisms. It also suggests that caspase-6 could be an important candidate target for the development of broad-spectrum, host-directed antiviral drugs. The existence of caspase-6 inhibitors developed for neurodegenerative diseases suggests their potential for repurposing to treat PRRSV infection.

However, this study has some limitations. First, the mechanism of caspase-6’s role in viral replication is not yet fully elucidated; its specific host and viral protein targets require further exploration. Second, the potential effects of the N protein cleavage fragments on other important immune pathways, such as NF-κB and the inflammasome, warrant subsequent in-depth investigation. Furthermore, although the PRRSV-D94A mutant strain showed good attenuation and immunogenicity, its safety in natural hosts, such as pregnant sows, and its immunomodulatory mechanisms in hosts at different developmental stages need to be systematically evaluated.

In conclusion, this study reveals for the first time a complex mechanism whereby PRRSV dynamically regulates host caspase-6 activity to target the 94th amino acid of the viral N protein, achieving immune evasion and promoting viral replication. It also establishes the molecular basis for using this site mutation in attenuated vaccine design. This discovery not only broadens the theoretical scope of virus–host interactions but also provides a new scientific basis and technical strategy for the future development of viral vaccines and host-directed antiviral drugs.

## MATERIALS AND METHODS

### Cells and viruses

Marc-145 and HEK-293T cells were cultured in Dulbecco’s modified Eagle’s medium (Biological Industries, Kibbutz Beit-Haemek, Israel) supplemented with 10% fetal bovine serum (Biological Industries). The cells were maintained at 37°C in a humidified 5% CO_2_ atmosphere. The PRRSV strains XH-GD (GenBank accession no. EU624117), VR2332 (GenBank accession no. AY150564), and NADC30 (GenBank accession no. JN654459) were propagated and titrated in Marc-145 cells and stored as aliquots at –80°C.

### Chemical modulators

The pan-caspase inhibitor, z-VAD-fmk, was obtained from Apexbio (A1902). The caspase-1-to-caspase-10 inhibitor sampler kit was purchased from R&D Systems. The caspase-6 inhibitor, Z-VEID-FMK, used for *in vitro* and *in vivo* experiments, was obtained from R&D Systems (FMK006) and APExBIO (A1923). The apoptosis enhancer STS (S5921) was obtained from Sigma. Filgotinib (JAK1 inhibitor) (HY-18300), Ruxolitinib (JAK1/2 inhibitor) (HY-50856), and IFNα-IFNAR-IN-1 hydrochloride (HY-12836A) were obtained from MedChemExpress. Poly(I:C) was from R&D Systems (4287/10).

### Antibodies

Anti-tubulin (#M20005s, Abmart), anti-caspase-6 (#9762, Cell Signaling), anti-FLAG (#20543-1-AP, Proteintech), anti-HA (#M20003s, Abmart), anti-FLAG (#80010-1-RR, Proteintech), anti-HA (#C29F4, Cell Signaling), anti-HA (M20003s, Abmart), anti-GFP (#50430-2-AP, Proteintech), anti-mCherry (#GTX128508, Gene Tex), anti-IRF 3 (#11312-1-AP, Proteintech), anti-actin (#66009-1-Ig, Proteintech), and PRRSV N (#JN0403, JNT) were purchased from the corresponding suppliers.

### RNA extraction and RT–qPCR

Cells were lysed in RL buffer and extracted using the MiniBEST Universal RNA Extraction Kit (TaKaRa). Viral RNA in the supernatant was extracted using the MiniBEST Viral RNA/DNA Extraction Kit (TaKaRa). RT–qPCR was performed using the Transcriptor First Strand cDNA Synthesis Kit and LightCycler 480 Master Mix from Roche. Data were collected through LightCycler96 (version SW1.1). All primer and probe sequences are provided in Table S1.

### TCID_50_ assays

Briefly, 96-well plates were seeded with 1 × 10^4^ Marc-145 cells per well and incubated overnight. A 100 μL aliquot of a 10-fold gradient viral dilution was used to inoculate the cells, followed by incubation for 1 h at 37°C. After discarding the viral solution, cells were washed with PBS. Each well received 100 μL of maintenance medium (DMEM with 2% FBS), and the incubation period was 5 days. The cytopathic effect (CPE) of the cells was monitored daily, and the TCID_50_ value was determined using the Reed–Muench method (43).

### siRNA knockdown

On-Targetplus caspase-6 siRNA was obtained from Dharmacon (L-004406-00-0005). Transfection of siRNA on MDMs was performed using Lipofectamine RNAiMAX (Thermo Fisher Scientific). Briefly, the cells were transfected with 50 nM caspase-6 siRNA for two consecutive days. At 24 h after the second siRNA transfection, the cells were collected in RIPA buffer for western blot analysis. In parallel, siRNA-transfected cells were challenged with PRRSV at 1 MOI for 1 h at 37°C. Following the inoculation, the cells were washed with PBS and incubated for 24 h. The virus copy number at 24 hpi was determined via RT–qPCR. All primer and probe sequences are provided in Table S2.

### IFA

Cells were inoculated in DMEM containing 10% FBS. After cell growth was stable, the plasmids were transfected into cells. After 24 h, cells were washed with cold PBS and then fixed with 4% paraformaldehyde. Permeabilization was performed using 0.1% Triton X-100, followed by blocking with 5% BSA. Cells were then incubated overnight with the appropriate primary antibodies, washed three times with PBS, and incubated with secondary antibodies (1:1,000) at room temperature for 1 h. After another three PBS washes, DAPI was added to each well for 5 min, followed by another three washes with PBS. Finally, images were obtained using a laser scanning confocal microscope.

### Western blot

Whole-cell lysates or IP samples were subjected to 10% sodium dodecyl sulfate-polyacrylamide gel electrophoresis. Subsequently, the resolved proteins were transferred to a nitrocellulose membrane. The membrane was blocked with 5% non-fat milk at 25°C for 1 h on a shaker, then incubated overnight at 4°C with a 1:1,000 dilution of anti-HA antibody (#M20003s), anti-FLAG antibody (#80010-1), and anti-tubulin antibody. Each membrane was then incubated with secondary antibodies in Tris-buffered saline with Tween at room temperature for 1 h. An Odyssey Infrared Imager (LI-COR Biosciences, Lincoln, NE, USA) was used to scan the membranes.

### Co-IP

The 293T cells were transfected with FLAG-plasmid together with HA-Caspase-6 plasmid. Cells transfected separately with each plasmid were used as negative controls. Cells were washed twice with cold PBS and lysed in lysis buffer with protease inhibitor cocktail (Beyotime, Shanghai, China) for 15 min, and then centrifuged at 12,000 × *g* at 4°C for 12 min. Co-IP was performed using dynabeads. The protein lysate was mixed with the bead–antibody complex and incubated for 4 h at room temperature (25°C) before washing the beads three times with lysis buffer. Bound proteins and 10% cell protein solution (Input) were detected via immunoblotting.

### Flow cytometry

Cells were harvested using 0.25 mM EDTA-trypsin after washing with PBS to remove residual serum. Digestion was terminated using serum-containing medium, and cells were gently resuspended to obtain a single-cell suspension. Cells were centrifuged at 1,000 × *g* for 5 min, washed once with PBS, and resuspended in 500 μL of 1× PBS. For apoptosis analysis, cells were stained with Annexin V-APC (Annexin V-APC Apoptosis Detection Kit) at room temperature in the dark for 30 min, with gentle pipetting every 10 min to ensure uniform staining. After staining, cells were washed, resuspended in PBS, filtered through a 300-mesh cell strainer, and analyzed using flow cytometry. For cell death analysis, cells were fixed in 4% paraformaldehyde at 4°C overnight, washed with PBS, and resuspended in 500 μL 1× PBS. Propidium iodide (PI, 50 μg/mL) was added, and cells were incubated at room temperature in the dark for 30 min, with periodic gentle mixing. Samples were filtered through a 300-mesh strainer and analyzed via flow cytometry to assess cell death.

### IFN-β luciferase reporter assays

IFN-β luciferase reporter assays were performed as previously described. Briefly, 293T cells were co-transfected with 500 ng of an IFN-β luciferase reporter plasmid, 10 ng of a transfection efficiency control plasmid (pNL1.1.TK, Promega), 1 μg of a PRRSV N plasmid, and 2 μg of a caspase-6 expression plasmid, with or without 2 μg Poly(I:C). After 24 h, cells were harvested, and luciferase activity was measured using the Dual-Luciferase Reporter Assay Kit (Vazyme) following the manufacturer’s instructions, with detection performed on a Victor X3 multi-label plate reader (Perkin-Elmer). Data were analyzed using the Kaleido (version 1.2) software. To investigate the point of action of the N fragments, IFNβ luciferase reporter plasmid pNL1.1.TK and PRRSV N (1–94) or PRRSV N (95–123) were co-transfected into 293T cells together with expression plasmids for RIG-IN, MAVS, or TBK1. At 24 h post-transfection, the cells were collected for luciferase measurement.

### Generation of mutant viruses via reverse genetics

Mutant viruses were generated by using the reverse genetics system as described previously (44). The rescued viruses were fully sequenced to ensure the absence of unwanted mutations.

### Animal pathogenicity experiment design

To study the pathogenicity of the mutant virus after infecting its natural host, PRRSV WT and PRRSV 94A were selected for challenge experiments. A total of 18 four-week-old piglets were used, randomly divided into three groups of six animals each. Animals were acclimated for 1 week, during which they tested negative for African swine fever virus, PRRSV, classical swine fever virus, and pseudorabies virus antigens. On day 0, each piglet was challenged with 2×10⁵ TCID₅₀/mL of the virus via both intranasal and intramuscular injections. On day 14, all piglets were euthanized, lung pathological changes were examined, and various tissue samples (lung, bronchial lymph nodes, and tonsils) were collected for further analysis (Fig. 8A).

### Serology

PRRSV-specific antibody (IgG) was detected using the PRRSV Antibody ELISA Test Kit (Beijing Jinnuo Baitai Biotechnology Co., Ltd.), according to the manufacturer’s instructions. An S/P ratio ≥ 0.4 was determined to be positive for the PRRSV antibody.

### Statistical analyses

Data are expressed as the mean ± SD. Statistical significance was determined using the Student’s two-tailed unpaired t test or ANOVA via the GraphPad Prism software (version 6.0, San Diego, CA). Differences between groups were considered significant when the *P*-value was <0.05 (*), <0.01 (**); “ns” indicates no significant difference.

## Data Availability

All data supporting the findings of this study are included in the article and its supplemental material. Raw data are available from the corresponding author upon reasonable request.

## SUPPLEMENTAL MATERIAL

Figure S1 jvi.00163-26-s0001.tif Co-localization of PRRSV viral protein and caspase-6 protein.

Figure S2 jvi.00163-26-s0002.tif Interaction between caspase-6 and PRRSV viral protein.

Figure S3 jvi.00163-26-s0003.tiff Interaction domains of N protein truncated fragments with karyopherins KPNA1, KPNA2, KPNA3, and KPNA4.

Figure S4 jvi.00163-26-s0004.tiff Knockdown of Caspase-1 and Caspase-5 significantly enhances PRRSV XH-GD replication.

Figure S5 jvi.00163-26-s0005.tiff Sequence alignment supporting the Sanger sequencing validation shown in Table S3.

Supplemental tables jvi.00163-26-s0006.docx Tables S1 to S3.

ASM does not own the copyrights to Supplemental Material that may be linked to, or accessed through, an article. The authors have granted ASM a non-exclusive, world-wide license to publish the Supplemental Material files. Please contact the corresponding author directly for reuse.
