# Supplementary figures and images for: Porcine reproductive and respiratory syndrome virus N protein-mediated viral replication enhancement via interaction with host caspase-6
Source: J Virol. 2026 Apr 14;100(5):e00163-26. doi: 10.1128/jvi.00163-26 (PMC13185617; doi:10.1128/jvi.00163-26)

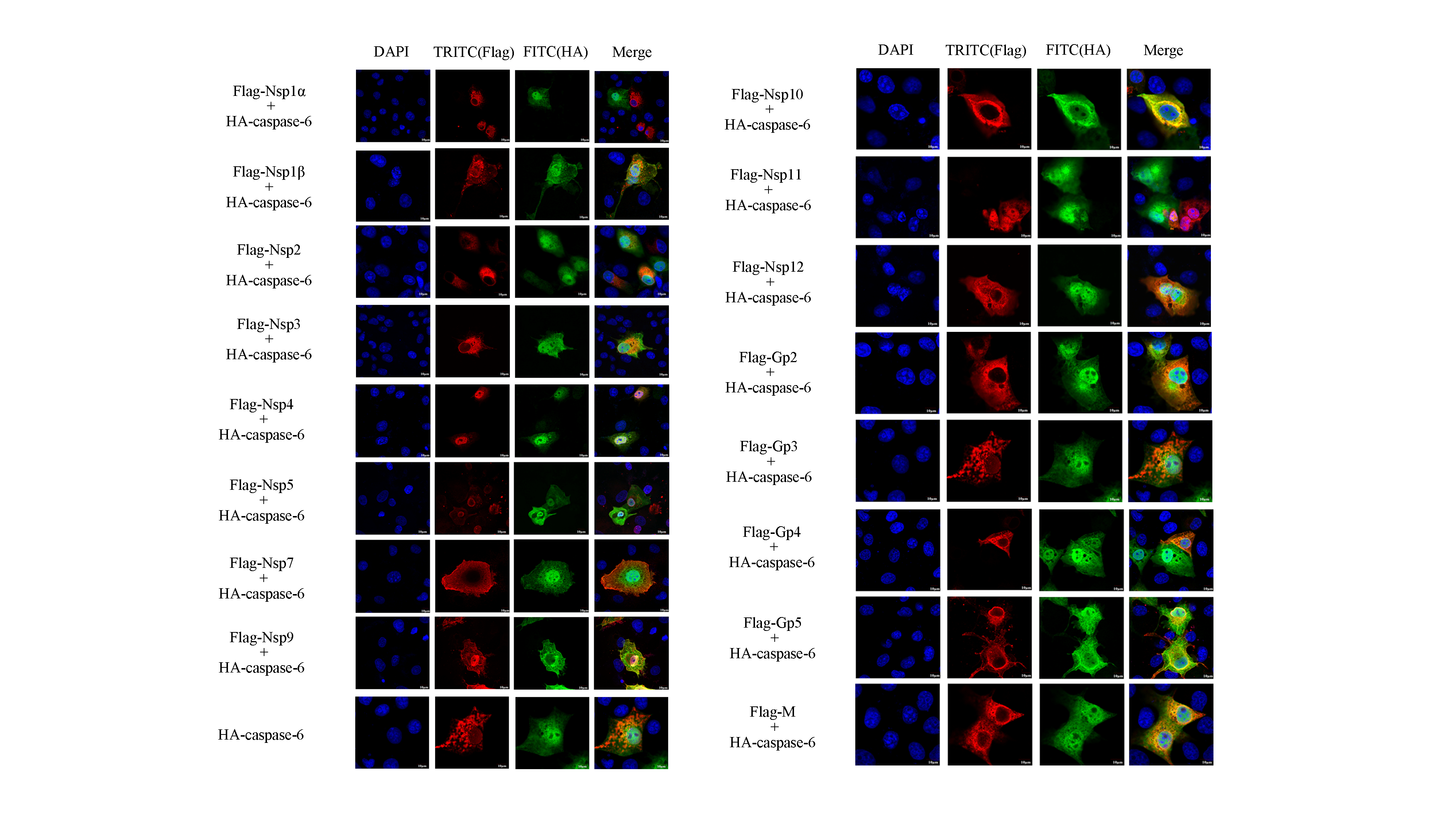

Supplement: Figure S1 — Co-localization of PRRSV viral protein and caspase-6 protein. [file jvi.00163-26-s0001.tif]

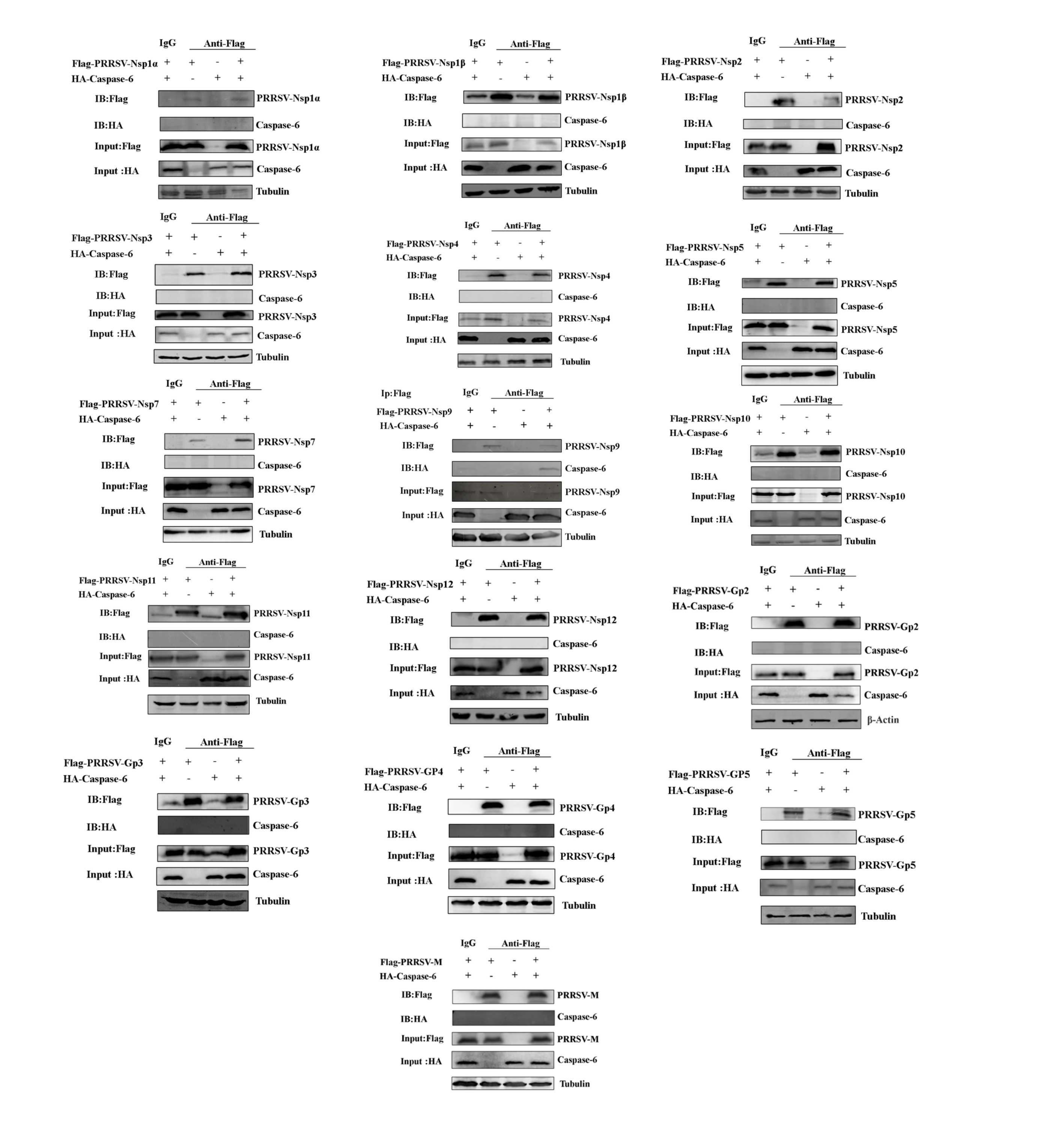

Supplement: Figure S2 — Interaction between caspase-6 and PRRSV viral protein. [file jvi.00163-26-s0002.tif]

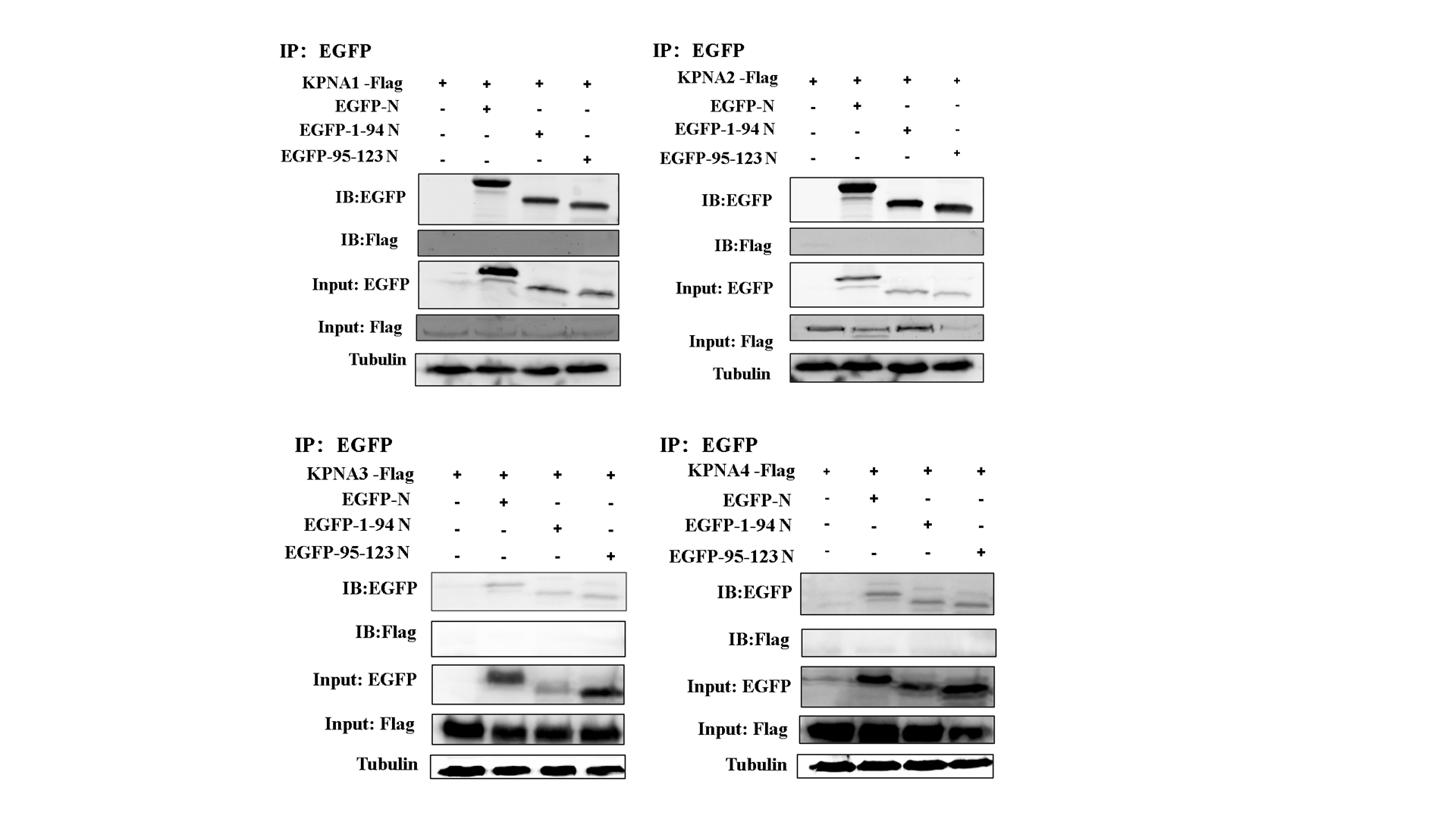

Supplement: Figure S3 — Interaction domains of N protein truncated fragments with karyopherins KPNA1, KPNA2, KPNA3, and KPNA4. [file jvi.00163-26-s0003.tiff]

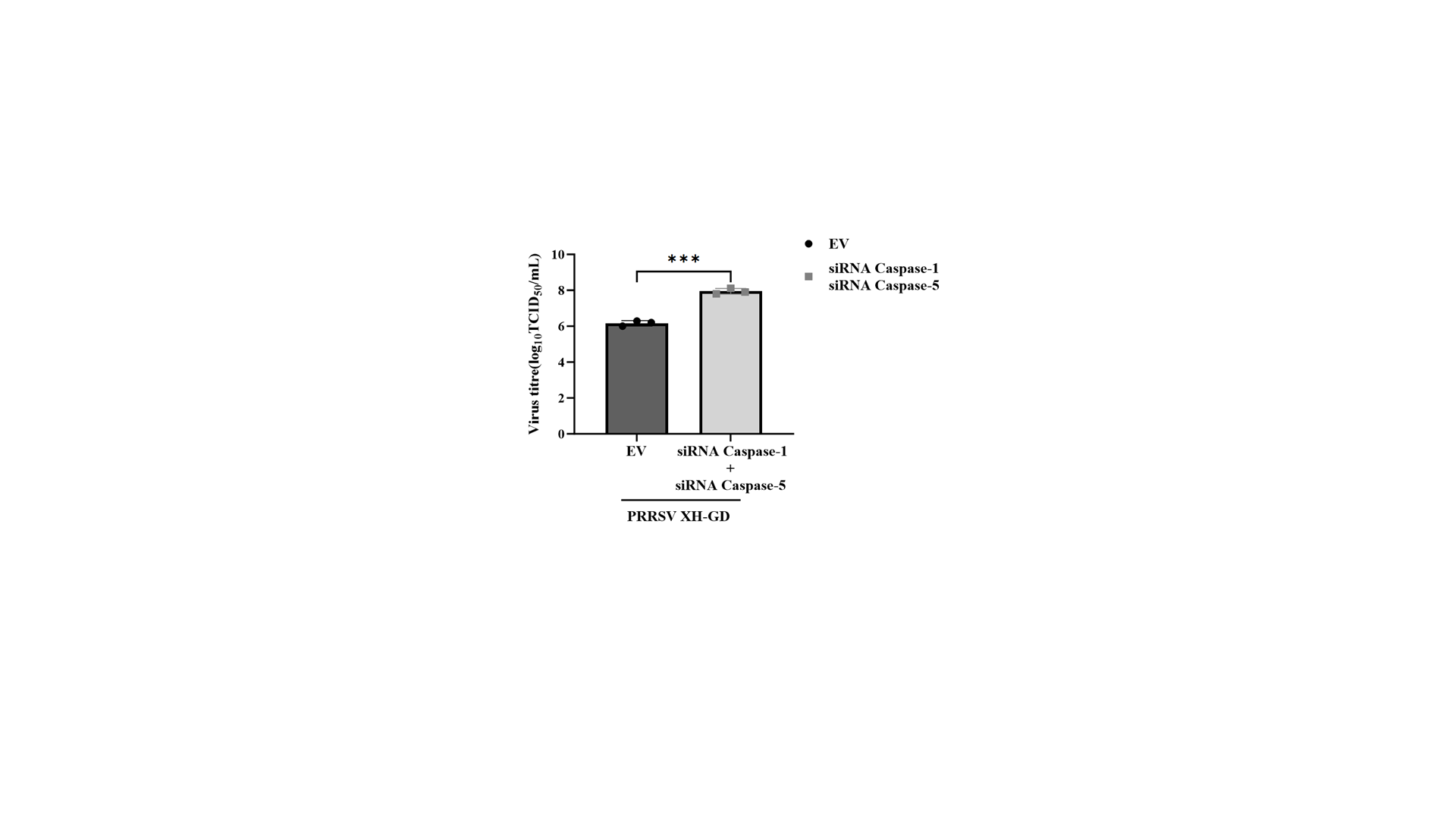

Supplement: Figure S4 — Knockdown of Caspase-1 and Caspase-5 significantly enhances PRRSV XH-GD replication. [file jvi.00163-26-s0004.tiff]

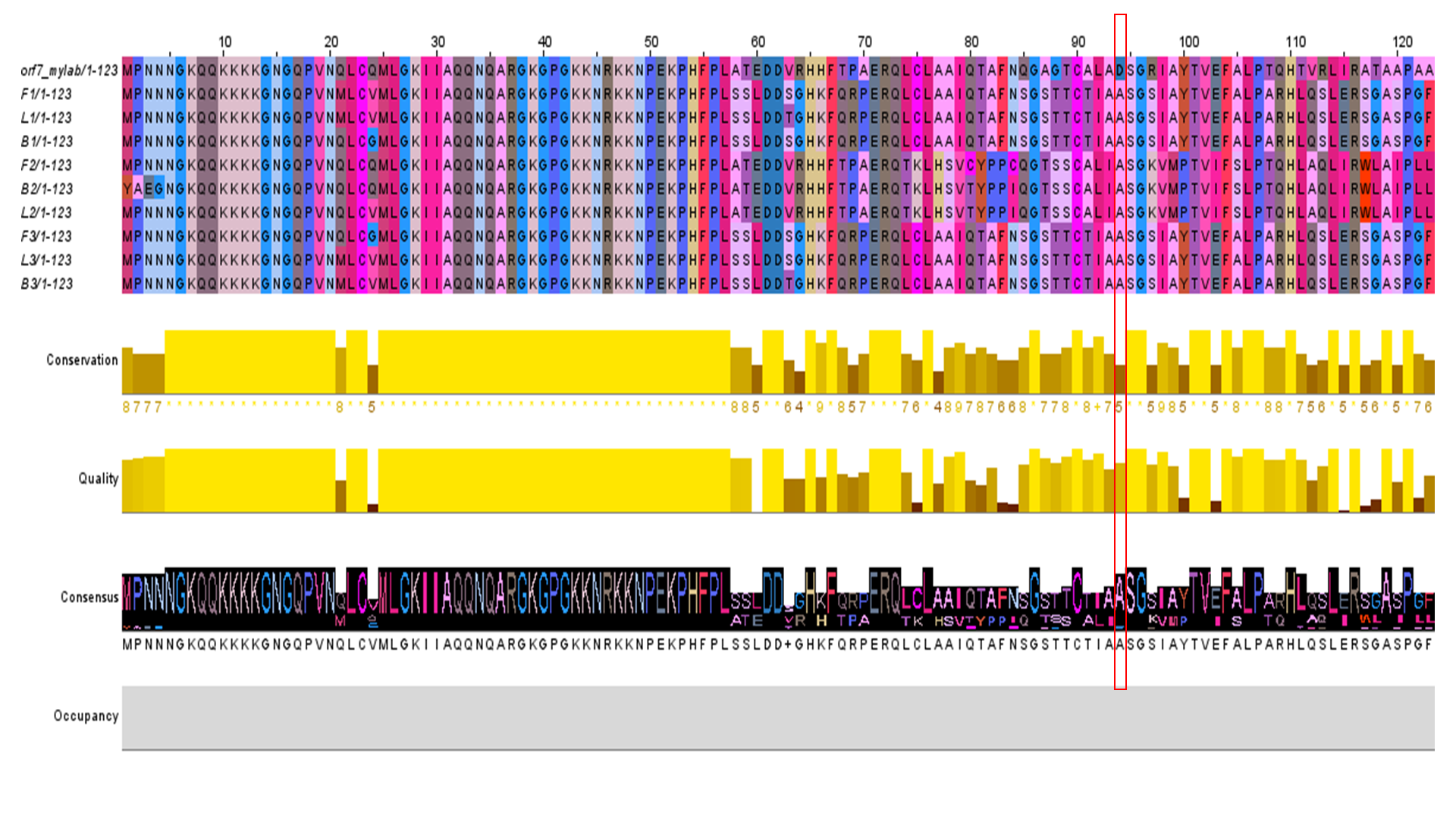

Supplement: Figure S5 — Sequence alignment supporting the Sanger sequencing validation shown in Table S3. [file jvi.00163-26-s0005.tiff]
