# Supplementary material for: Porcine reproductive and respiratory syndrome virus N protein-mediated viral replication enhancement via interaction with host caspase-6
Source: J Virol. 2026 Apr 14;100(5):e00163-26. doi: 10.1128/jvi.00163-26 (PMC13185617; doi:10.1128/jvi.00163-26)
Supplement: Supplemental tables — Tables S1 to S3. [file jvi.00163-26-s0006.docx]

**Supplementary Material**

**Supplementary Figure 1.** Co-localization of PRRSV viral protein and caspase-6 protein.

**Supplementary Figure 2.** Interaction between caspase-6 and PRRSV viral protein.

**Supplementary Figure 3.** Interaction domains of N protein truncated fragments with karyopherins KPNA1, KPNA2, KPNA3, and KPNA4.

**Supplementary Figure 4.** Knockdown of Caspase-1 and Caspase-5 significantly enhances PRRSV XH-GD replication.

**Supplementary Figure 5.** Sequence alignment supporting the Sanger sequencing validation shown in Supplementary Table 3.

**Supplementary Table 1.** List of RT-qPCR primers.

| Primer Name | Primer sequence（5′ ~ 3 ′） |
| --- | --- |
| IFN-β-F | AGGAGGACGCCGCATTGAC |
| IFN-β-R | GATGATAGACATTAGCCAGGAGGTTC |
| IFIT1-F | CCAACCAAGCAAGTGTGAGGAGTC |
| IFIT1-R | CTTCTGCCAGTCTGCCCATGTG |
| IFIT2-F | AGGCGAAGAGGAAGGTGAAGGAG |
| IFIT2-R | TGCTGCACTGCGAAGTACATCTG |
| IFIT3-F | ACGCCTGGGTCTACTATCACTTGG |
| IFIT3-R | CACTTCAGTTGTGTCCACCCTTCC |
| OAS1-F | GCGAGTTCTCCACCTGCTTCAC |
| OAS1-R | ACTAGGCGGATGAGGCTCTTGAG |
| IFITTM3-F | CCAAACCTTCTTCTCTCCTGTCAAC |
| IFITTM3-R | AGGTCTCGCTGCGGATGTG |

**Supplementary Table 2.** Oligonucleotide sequences used in siRNAs.

| Primer Name | Primer sequence（5 ′ ~ 3 ′） |
| --- | --- |
| Caspase-1-1 | GCAUGACAAUGCUGCUACA(dT)(dT) |
| Caspase-1-2 | UGUAGCAGCAUUGUCAUGC(dT)(dT) |
| Caspase-3-1 | GAUUGUUGUAGAAGUCUAA(dT)(dT) |
| Caspase-3-2 | UUAGACUUCUACAACAAUC(dT)(dT) |
| Caspase-4-1 | GGACUAUAGUGUGGAUGUA(dT)(dT) |
| Caspase-4-2 | UACAUCCACACUAUAGUCC(dT)(dT) |
| Caspase-5-1 | CAAGGUAGACUCUGUGCUA(dT)(dT) |
| Caspase-5-2 | UAGCACAGAGUCUACCUUG(dT)(dT) |
| Caspase-6-1 | GAUGCAGUAGAUAAUCAGA(dT)(dT) |
| Caspase-6-2 | UCUGAUUAUCUACUGCAUC(dT)(dT) |
| Caspase-7-1 | GAUGCUAAUCCUCGAUAUA(dT)(dT) |
| Caspase-7-2 | UAUAUCGAGGAUUAGCAUC(dT)(dT) |
| Caspase-8-1 | GAUACUGUCUGAUCAUCAA(dT)(dT) |
| Caspase-8-2 | UUGAUGAUCAGACAGUAUC(dT)(dT) |
| Caspase-9-1 | CGACCAGCUGGAUGCUGUA(dT)(dT) |
| Caspase-9-2 | UACAGCAUCCAGCUGGUCG(dT)(dT) |
| Caspase-10-1 | CGAUGAUGUGAGUCGACGA(dT)(dT) |
| Caspase-10-2 | UCGUCGACUCACAUCAUCG(dT)(dT) |
| Caspase-11-1 | GGACGACAACUACAACUUA(dT)(dT) |
| Caspase-11-2 | UAAGUUGUAGUUGUCGUCC(dT)(dT) |
| Caspase-12-1 | CGAUGACACCAUCUUCGAA(dT)(dT) |
| Caspase-12-2 | UUCGAAGAUGGUGUCAUCG(dT)(dT) |
| Caspase-14-1 | GGCCUGUCGAGGAGAACAA(dT)(dT) |
| Caspase-14-2 | UUGUUCUCCUCGACAGGCC(dT)(dT) |

**Supplementary Table 3.** Sanger Sequencing Validation Results**.**

| Group | Sample Name | 94th Amino Acid | Sample Name | 94th Amino Acid | Sample Name | 94th Amino Acid |
| --- | --- | --- | --- | --- | --- | --- |
| PRRSV-94A | F1 (Lung) | GCT (Ala) | F2 (Lung) | GCT (Ala) | F3 (Lung) | GCT (Ala) |
|  | L1 (Lymph Node) | GCT (Ala) | L2 (Lymph Node) | GCT (Ala) | L3 (Lymph Node) | GCT (Ala) |
|  | B1 (Serum) | GCT (Ala) | B2 (Serum) | GCT (Ala) | B3 (Serum) | GCT (Ala) |
